# Eco‐Geography Reverses Dominant AMR Reservoirs in *Klebsiella pneumoniae*: Integron‐Rich Mobilomes and Cross‐Niche Connectivity

**DOI:** 10.1002/advs.75537

**Published:** 2026-05-06

**Authors:** Hui Lin, Biao Tang, Hao Xu, Jiajie Qian, Feifan Shi, Junhui Zhao, Xiaorui Mao, Xiaohe Hu, Ruishan Liu, Wenhong Liu, Xiawei Jiang, Beiwen Zheng, Guoping Zhao

**Affiliations:** ^1^ State Key Laboratory for Quality and Safety of Agro‐Product Zhejiang Provincial Key Laboratory of Agricultural Microbiomics Institute of Environment Resource, Soil and Fertilizer Zhejiang Academy of Agricultural Sciences Hangzhou P. R. China; ^2^ Key Laboratory of Systems Health Science of Zhejiang Province School of Life Science Hangzhou Institute for Advanced Study University of Chinese Academy of Sciences Hangzhou P. R. China; ^3^ State Key Laboratory for Diagnosis and Treatment of Infectious Diseases National Clinical Research Center for Infectious Diseases National Medical Center for Infectious Diseases Collaborative Innovation Center for Diagnosis and Treatment of Infectious Diseases The First Affiliated Hospital Zhejiang University Hangzhou P. R. China; ^4^ Department of Gastrointestinal Surgery The First Affiliated Hospital College of Medicine Zhejiang University Hangzhou P. R. China; ^5^ Xianghu Laboratory Hangzhou P. R. China; ^6^ Department of Clinical Laboratory The Second Affiliated Hospital of Wannan Medical College Wuhu P. R. China; ^7^ School of Basic Medical Sciences Zhejiang Chinese Medical University Hangzhou P. R. China; ^8^ Yuhang Institute of Medical Science Innovation and Transformation Hangzhou P. R. China; ^9^ Jinan Microecological Biomedicine Shandong Laboratory Jinan P. R. China; ^10^ Department of Microbiology School of Life Sciences Fudan University Shanghai P. R, China

**Keywords:** antimicrobial resistance, cross‐niche connectivity, eco‐geography, integron, *Klebsiella pneumoniae*, one health, surveillance

## Abstract

Multidrug‐resistant *Klebsiella pneumoniae* is a global health threat circulating across humans, animals, food, and environments. Despite increasing One Health surveillance, it remains unclear whether the dominant antimicrobial resistance (AMR) reservoirs in *K. pneumoniae* are universal or depend on regional ecological context. Here, a synchronized One Health survey in Pingguo, China (5384 samples) is integrated with parallel cross‐niche datasets (Italy, Ghana) and a 69184 public genome collection, mapping AMR ecology via network metrics and lineage‐aware mixed models. In Pingguo, high‐risk determinants are enriched in humans, whereas overall AMR burdens and multidrug resistance platforms are enriched outside humans. Across the public dataset, AMR reservoir dominance varies eco‐geographically, with nonhuman isolates carrying higher AMR gene burdens in the China subset (mean +4.5 per isolate), largely due to animal and environmental isolates, while human isolates carry higher burdens in the U.S. and Europe subsets. Lineage explains 44.8% of the burden difference. Remaining within‐lineage differences, reservoir‐matched integron enrichment, and region‐specific connectivity suggest additional horizontal gene transfer‐related contributions. This is supported in Pingguo by a highly structured cross‐niche plasmid‐sharing network shaped by specific plasmid groups and successful lineages. Overall, dominant AMR reservoirs are eco‐geographically contingent. Integron load and cross‐niche connectivity provide indicators for tiered, region‐tailored surveillance.

## Graphical abstract

Dominant antimicrobial resistance reservoirs in *Klebsiella pneumoniae* vary across eco‐geographic settings rather than following a universal pattern. Integrated One Health and global genomic analyses show that lineage structure, integron load, and cross‐niche connectivity shape whether AMR burden accumulates primarily in human or nonhuman compartments.

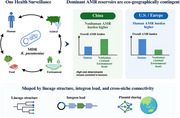

## 1 Introduction

Antimicrobial resistance (AMR) is an escalating global health problem that threatens effective treatment and carries substantial socioeconomic costs [1]. Multidrug‐resistant *Klebsiella pneumoniae* is a primary driver of the global AMR crisis [2, 3]. Currently, the worldwide expansion of its carbapenem‐resistant lineages, coupled with an increasing co‐occurrence of resistance and virulence determinants, is rapidly narrowing therapeutic options and magnifying clinical risks [1, 4].

A defining feature of *K. pneumoniae* is its broad ecology. The *K. pneumoniae* species complex (KpSC) is ubiquitous across clinical and community settings, as well as nonhuman reservoirs, including animal, food, and environmental compartments [3]. Consistently, overlaps in clonal lineages, AMR determinants, and virulence profiles have been documented between human and nonhuman *K. pneumoniae* isolates [5, 6]. Some nonhuman isolates also show pathogenic potential in human infection models [5, 7, 8].

Despite increasing adoption of One Health frameworks [9, 10, 11], a central unresolved question remains: are dominant reservoirs driving the spread of AMR in *K. pneumoniae* universal, or contingent on regional ecological context? Most cross‐sectoral genomic studies for *Klebsiella* have been conducted in high‐income settings such as in the U.S. and Europe, where clinical compartments were often considered as the primary AMR reservoir for *K. pneumoniae* [12, 13, 14, 15]. However, extrapolating such patterns globally may obscure alternative ecological configurations in which nonhuman reservoirs may act not merely as passive sinks, but as upstream amplifiers of transmissible resistance platforms.

China provides an important setting in which to test regional portability. *K. pneumoniae* ranks among the most frequently detected clinical pathogens in China [16]. Numerous genomic studies [17, 18, 19, 20] have made China a major contributor to publicly available *K. pneumoniae* genome sources (Figure S1, Supporting File 2). However, integrated cross‐sector genomic surveys that synchronously sample humans, animals, food, and the environment remain scarce in China. This gap matters because China's history of intensive antibiotic usage and dense human–animal–environment interfaces may fundamentally influence reservoirs, transmission routes, and selection pressures acting on AMR determinants [21, 22, 23, 24].

Here, we hypothesize that AMR reservoir dominance in *K. pneumoniae* is eco‐geographically structured. Specifically, we propose that lineage composition and mobilome intensity jointly determine whether resistance accumulation is primarily human‐centered or amplified across nonhuman niches. To test this, we integrated a synchronized county‐level One Health sampling in China with cross‐regional genomic datasets from Europe (Italy) [12] and Africa (Ghana) [25], together with 69184 publicly available genomes from 126 countries/regions. By quantifying integron‐rich mobilomes and cross‐niche connectivity, two metrics computable from routine genomic surveillance, we aim to define scalable indicators capable of stratifying regional AMR ecologies and informing tiered surveillance priorities. To ensure analytical robustness, we implemented sample‐size‐matched resampling and independent validation against a previously constructed, curated, and nonredundant global dataset [26], thereby minimizing biases arising from uneven sampling density and dataset redundancy.

## 2 Results

### 2.1 *K. pneumoniae* Populations in Pingguo Show Strong Ecological Structuring

**FIGURE 1:**
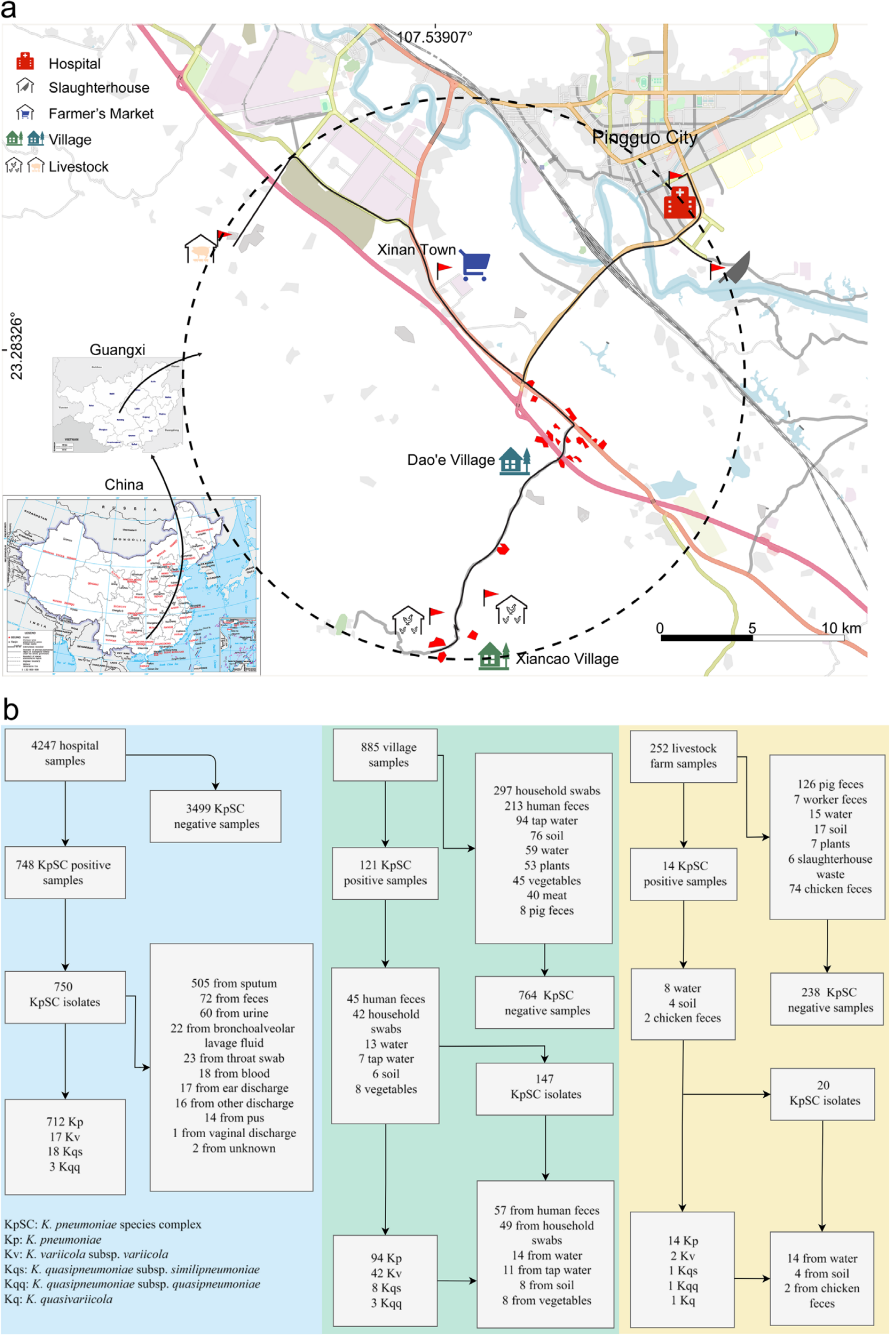
Summary of the sampling effort. (a) Geographical summary of the sampling areas. (b) Flowchart showing the number of initial samples, retained samples, and *Klebsiella pneumoniae* species complex (KpSC) species after each processing step. The base map for the main panel contains data from OpenStreetMap contributors (ODbL; https://www.openstreetmap.org/copyright). The China and Guangxi inset maps were obtained from the Standard Map Service of the Ministry of Natural Resources of China (http://bzdt.ch.mnr.gov.cn/).

**FIGURE 2:**
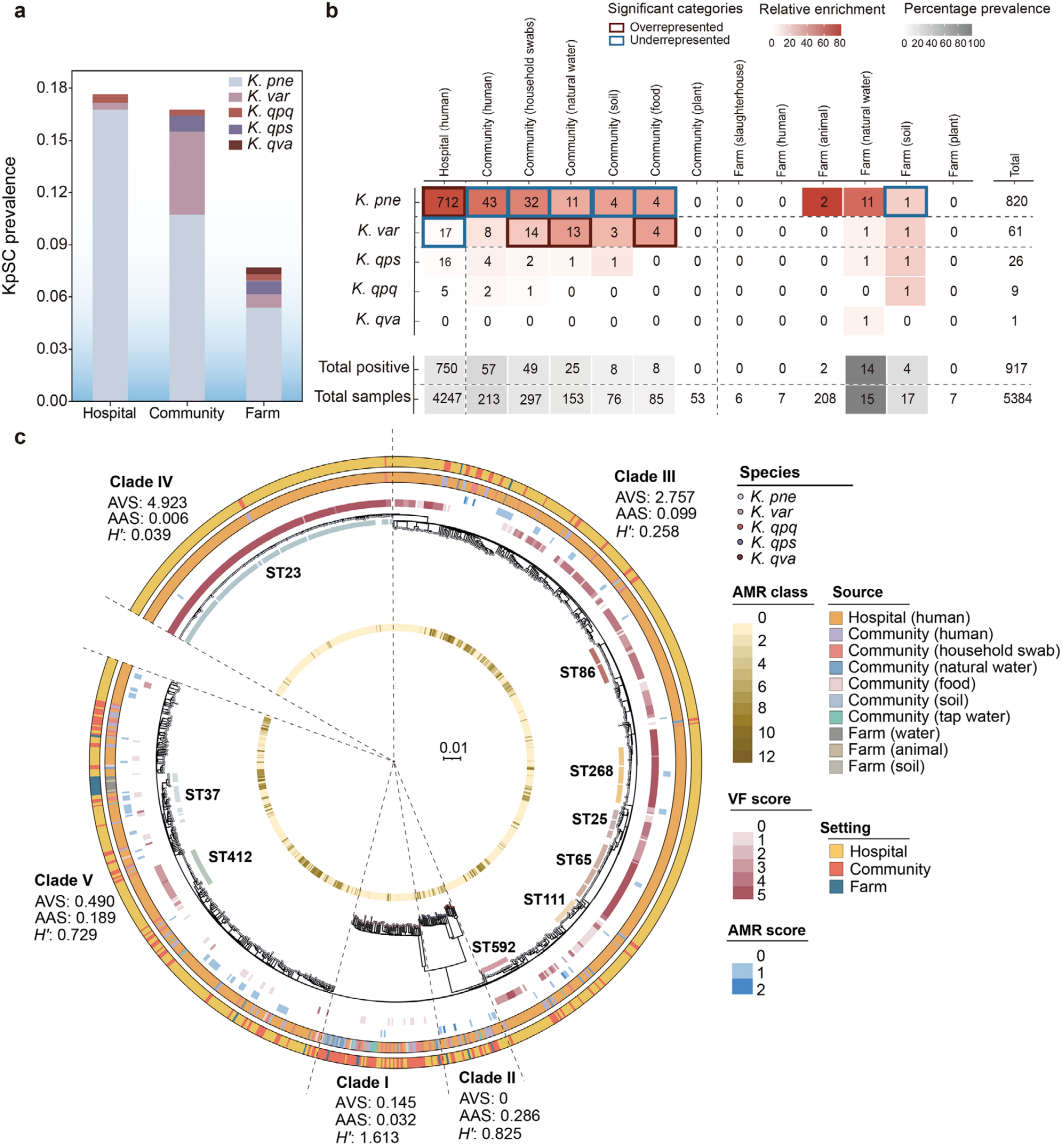
Ecological niche structures the *Klebsiella pneumoniae* species complex (KpSC). (a) Stacked bars show KpSC prevalence across three major settings (total samples, *n* = 5384; KpSC‐positive isolates, *n* = 917). (b) Distribution matrix of KpSC species. Cell borders mark significant over‐ (dark red) or under‐representation (dark blue) from permutation tests (*p* < 0.05; Data S10). Grey marginals summarize per‐species (rows) and per‐source totals (columns). (c) Neighbor‐joining tree of 917 representative isolates. Concentric rings annotate antimicrobial resistance (AMR) class, virulence (VF) score, AMR score, source, and setting (from inner to outer). Text next to major clades reports mean virulence score (AVS), mean AMR score (AAS), and the Shannon diversity index (*H'*). Major STs are labeled. *K. pne*: *Klebsiella pneumoniae* sensu stricto; *K. var*: *Klebsiella variicola* subsp. *Variicola*; *K. qpq*: *Klebsiella quasipneumoniae* subsp. *quasipneumoniae*; *K. qps*: *Klebsiella quasipneumoniae* subsp. *similipneumoniae*; *K. qva*: *Klebsiella quasivariicola*.

A total of 5384 samples were collected from 167 locations across hospitals, communities, and farms in Pingguo, a county‐level city in southern China (Figure 1a). From human, environment, animal, and food sources, we recovered 917 KpSC isolates [3] (Figure 1b). KpSC prevalence varies across ecological niches. Prevalence is highest in human‐associated settings, including hospitals (17.7%, 748/4247 samples) and communities (13.7%, 121/885 samples), while relatively lower on farms (5.6%, 14/252 samples) (Figure 2a).

KpSC populations show strong ecological structuring. *Klebsiella variicola* subsp. *variicola* is enriched in environmental samples, while *K. pneumoniae* sensu stricto (*K. pneumoniae*) is overrepresented in clinical human isolates (Permutation test, adjusted *p* < 0.001; Figure 2b). Phylogenetic analysis groups these isolates into five major phylogenetic clades, including three within *K. pneumoniae* (clades III, IV, V; Figure 2c). Clade IV, a hospital‐associated lineage dominated by ST23, consists almost exclusively of highly virulent but susceptible isolates. In contrast, clades III and V span ten source types, show moderate virulence scores, and contain sublineages with high AMR scores.

Ecological structuring persists at the sequence type (ST) level. Among 820 *K. pneumoniae* isolates, 239 STs were identified, with ST23, ST268, ST37, ST412, ST65, and ST86 being the most frequent (Figure S2, Supporting File 2). ST composition differs significantly across settings (hospital/farm/community) and between human and nonhuman sources (Pearson's χ^2^ tests, both *p* < 0.001, unbalanced full dataset). These associations remain robust after repeated down‐sampling to balance source size (100 iterations). Hypervirulent lineages ST23 and ST65 are overrepresented in hospital samples (Permutation test, adjusted *p* < 0.001), while ST37 isolates are enriched in community samples (Permutation test, adjusted *p* = 0.019).

### 2.2 Clonal Sharing in Pingguo is Confined Within Settings and Frequent in the Community

**FIGURE 3:**
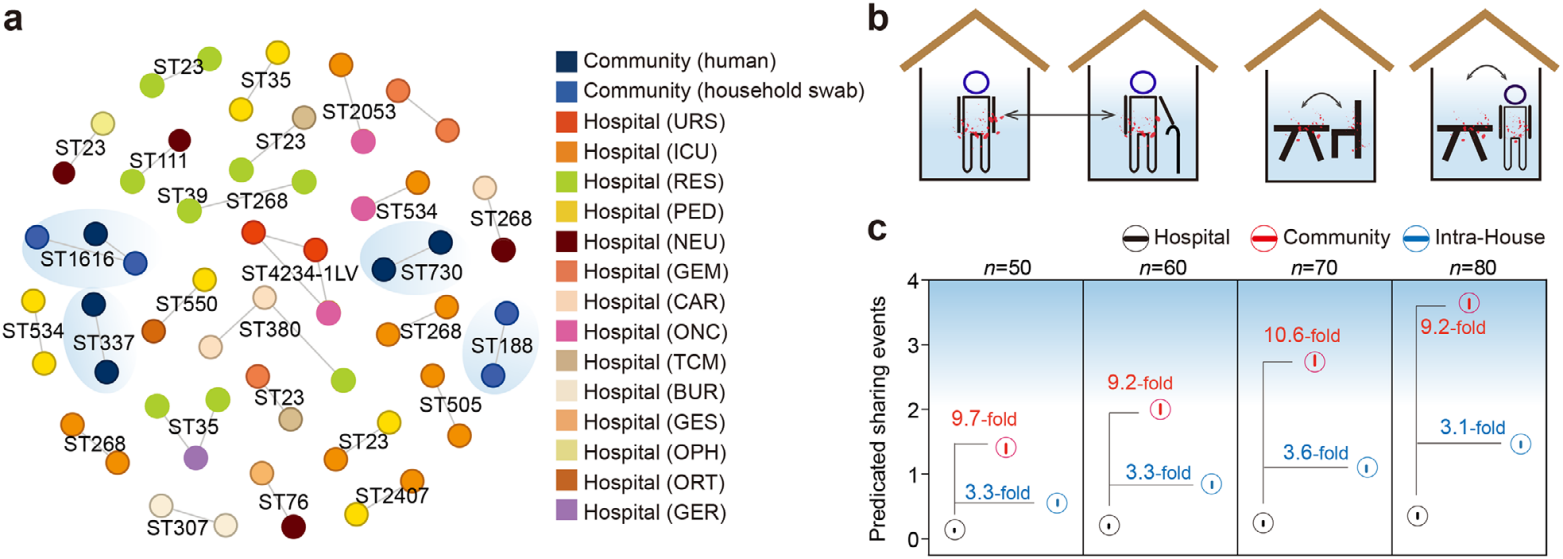
Clonal sharing events of *Klebsiella pneumoniae* across sampling contexts. (a) Force‐directed (Fruchterman‐Reingold) network of genomically linked isolates, defined as pairs by ≤10 core‐genome SNPs. Within‐sample duplicates were removed, and multiple links between the same pair of samples were collapsed to a single edge. Node colors denote sampling sources (legend). (b) Schematic of community contexts showing inter‐household person‐to‐person links and intra‐household person‐environment links. (c) Expected numbers of clonal‐sharing events by sampling context (patients sampled at the hospital, community‐wide, and intra‐household) were estimated using size‐matched resampling (*n* = 50, 60, 70, 80; 1000 iterations each). Vertical lines indicate 95% bootstrap confidence intervals. Abbreviations: URS, Urology Department; ICU, Intensive Care Unit; RES, Respiratory Department; PED, Pediatric Department; NEU, Neurology Department; GEM, General Medicine Department; CAR, Cardiovascular Department; ONC, Oncology Department; TCM, Traditional Chinese Medicine Department; BUR, Burn Department; GES, General Surgery Department; OPH, Ophthalmology Department; ORT, Orthopedic Department; GER, Geriatric Medicine Department.

Although 30 STs are shared across settings in Pingguo (Figure S2, Supporting File 2), single‐nucleotide polymorphism (SNP)‐based analyses detected putative clonal sharing only within, not between, settings (Figure S3, Supporting File 2). Applying a 10‐SNP threshold to define genomic similarity as a proxy for recent transmission [12, 27], we identified 27 clonal sharing links in hospital including 42 patient isolates and 5 links involving 9 community isolates (Figure 3a). Clonal sharing among hospital isolates occurs both within and between departments, suggesting potential nosocomial transmission, with repeated sharing events observed for ST23 and ST268. These lineages are prevalent locally and are commonly associated with hypervirulence [26]. In the community, genomic links connect elderly individuals, adults, and their household environments, representing both intra‐ and inter‐household transmission (Figure 3b).

To compare the transmission potential across settings with different sampling densities, we employed two normalization strategies. First, a simple normalization method showed that the proportion of isolates involved in clonal sharing was higher in the community than in hospitals (9.6%, 9/94 vs. 5.9%, 42/712). This trend was confirmed in a more robust iterative subsampling analysis. At matched sample sizes, the community setting consistently exhibits a higher frequency of sharing links per resample than the hospital setting (e.g., mean 1.40 vs. 0.13 events at *n* = 50; Mann–Whitney *U* test, *p* < 0.001) (Figure 3c). This community‐skewed signal persists after removing all intra‐household links. Inter‐household links alone (0.57–1.46 across matched sizes *n* = 50–80) remain ∼3.2‐fold higher than those observed among hospital‐sampled patients, suggesting extensive clonal transmission outside clinical settings.

### 2.3 Ecological Decoupling of Overall AMR Burden and Clinically High‐Risk Determinants in Pingguo

**FIGURE 4:**
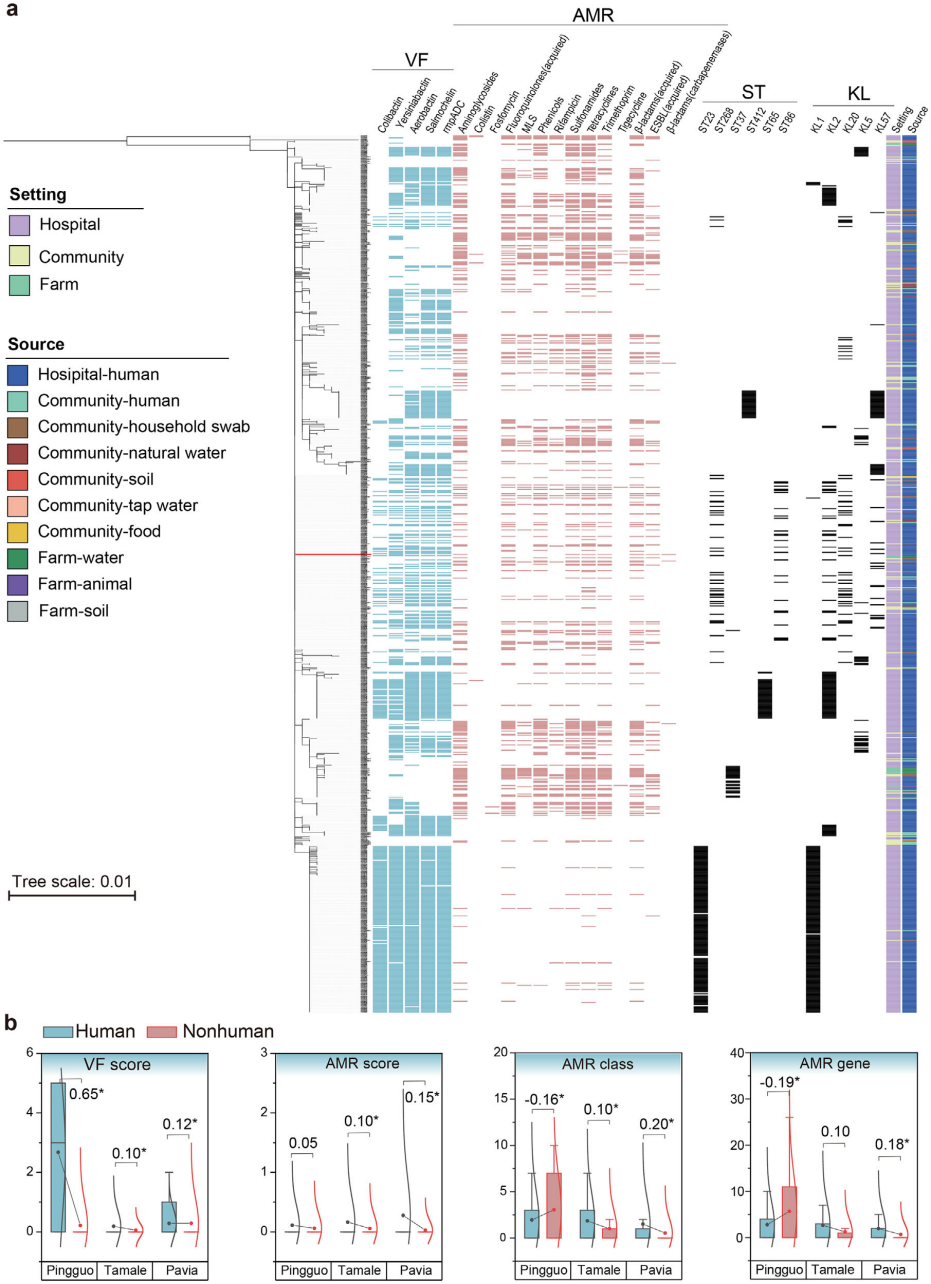
Phylogenomic context of *Klebsiella pneumoniae*. (a) Core‐genome maximum‐likelihood phylogeny of Pingguo isolates (left) with heatmaps showing: (i) virulence (VF)‐associated loci, including colibactin, yersiniabactin, aerobactin, salmochelin, and rmpADC (light blue); (ii) antimicrobial resistance (AMR) categories based on acquired determinants (light red); (iii) sequence types, including major clonal groups (ST; black bars); (iv) capsule loci, including predominant K‐locus types (KL; black bars); and (v) ecological metadata, including sampling setting and source (outer color strips). The red line marks the reference genome (GCA_000240185.2). (b) Human to nonhuman contrasts in Pingguo (this study), Tamale (Ghana), and Pavia (Italy) for VF scores, AMR scores, and AMR class and gene burdens. Effect sizes are Cliff's δ (human vs. nonhuman) with asterisks indicating *p* < 0.05 (Mann–Whitney U test).

In Pingguo, 41.0% (336/820) of *K. pneumoniae* isolates are classified as hypervirulent, defined by the presence of aerobactin and salmochelin together with truncated RmpA2 [25]. This prevalence far exceeds that reported in similar cross‐niche studies from Italy (0.09%) [12] and Ghana (0.54%) [25]. Virulence genes are concentrated in human isolates, which exhibit significantly higher mean virulence score (Kleborate scale 0–5) [26] than nonhuman isolates (Figure 4). Almost all the hypervirulent isolates are hospital‐derived, with only two from community humans and one from the community environment (Figure 4a). This niche partitioning of hypervirulence is consistent with findings from Italy [12] and Ghana [25], suggesting a relatively conserved epidemiological pattern.

Analysis of AMR metrics reveals distinct ecological distributions for overall AMR burden and AMR score, the latter being based on the presence of extended‐spectrum β‐lactamase genes, carbapenemase genes, and colistin resistance. On one hand, nonhuman isolates in Pingguo harbor significantly more AMR gene (mean 5.7 vs. 2.8, *p* = 0.003) and class burdens (mean 3.1 vs. 1.9, *p* = 0.009) than human isolates (Figure 4b). This is corroborated by phenotypic antimicrobial susceptibility testing, showing resistance to a greater number of antimicrobial classes in nonhuman isolates (nonhuman vs. human: 4.1 vs. 1.9 resistance classes; Table S1). On the other hand, AMR genes targeting last‐resort antibiotics, including carbapenemases and colistin resistance determinants, are restricted to human clinical isolates, aligning with higher AMR scores in human isolates (Figure 4a). Surveys from Italy [12] and Ghana [25] show similar AMR scores but more AMR genes and classes in human isolates, underscoring regional heterogeneity (Figure 4b). Overall, nonhuman reservoirs in Pingguo are enriched for overall AMR burden, whereas carbapenemase genes, colistin resistance, and hypervirulence remain concentrated in human clinical isolates.

### 2.4 Public Genomes Reveal Eco‐Geographical Contrasts in AMR Reservoir Structure

**FIGURE 5:**
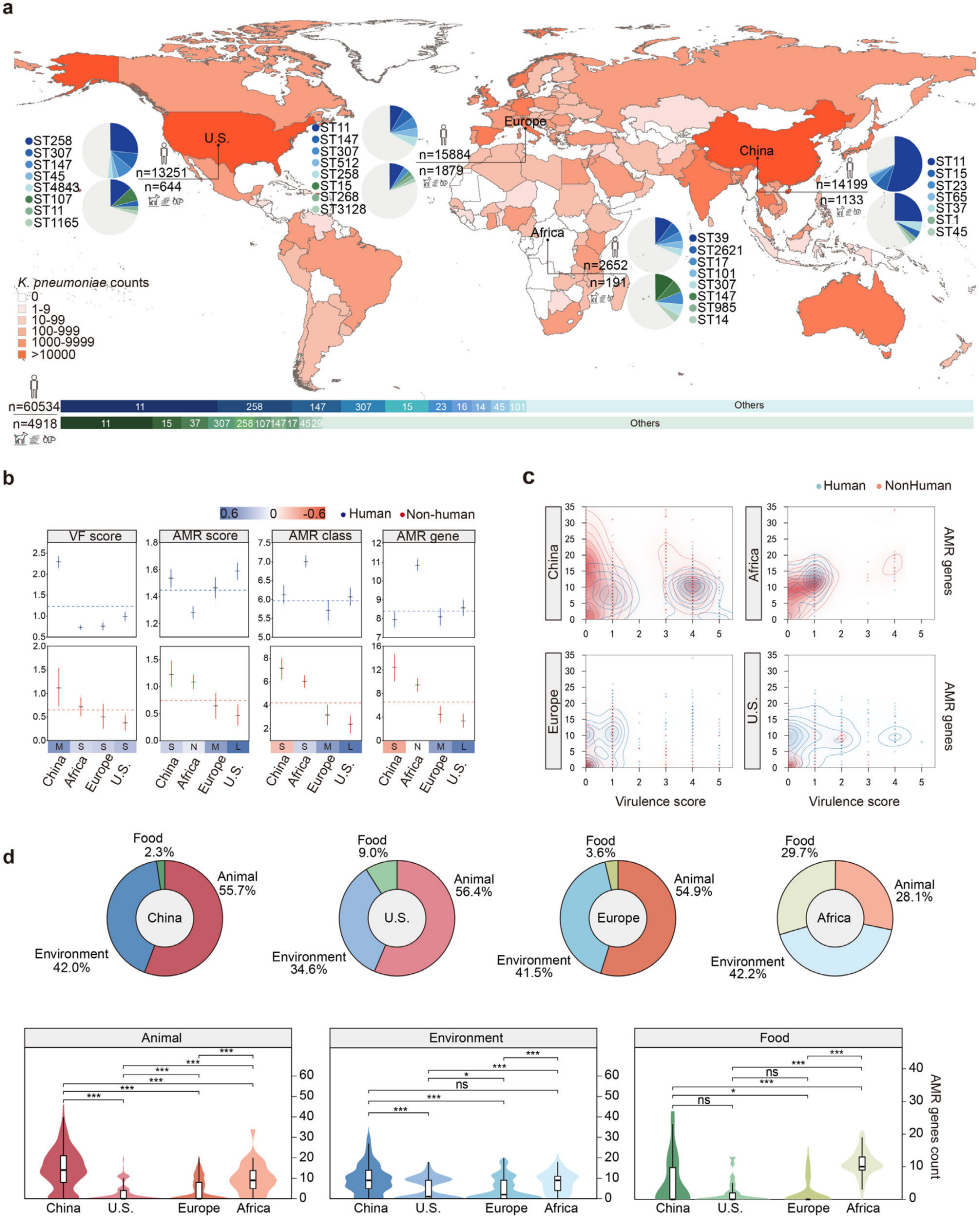
Global diversity, antimicrobial resistance (AMR), and virulence profiles of *K. pneumoniae* across ecological niches. (a) Geographic distribution of regional lineage dominance. Country shading indicates genome sampling density (white = no data). Pies show the most prevalent STs by sampling source (human = top pie, nonhuman = bottom); sample sizes (*n*) are indicated. Horizontal stripes summarize global proportions of dominant STs (labels denote STs). Base map: GADM (https://gadm.org/). (b) Virulence and AMR metrics by region and source. Points show means ± 95% CI for human (blue) and nonhuman (red) isolates; dashed bands mark the global mean. Human isolates consistently have higher virulence scores (VF score) than nonhuman isolates (typically with small (S)‐medium (M) effects). AMR gene burden diverges by region: higher in human isolates in Europe (medium effect size, M) and the U.S. (large effect size, L), but higher in nonhuman isolates in China (small effect size, S). The heatmap reports the Cliff's δ effect size (human vs. nonhuman): N, negligible <0.147; S, small 0.147–0.33; M, medium 0.33–0.474; L, large > 0.474. (c) 2D density contours of aggregate AMR and virulence traits by region and source (blue = human, red = nonhuman). China and Africa show human‐nonhuman homogenization, in contrast to niche segregation in the U.S. and Europe. (d) Source composition and AMR gene burden in nonhuman datasets. Pie charts show the proportions of animal, environment, and food sources. Violin plots compare AMR gene counts per isolate across regions within each source category. Brackets indicate pairwise Mann–Whitney U tests (***p < 0.001; ns, not significant).

To contextualize our local findings, we analyzed 69184 publicly available *K. pneumoniae* genomes (3170 STs) (Figure 5a). Across the global public dataset, ST distributions differ by source (human vs. nonhuman; Pearson's χ^2^, *p* < 0.001); this signal persists under balanced resampling. Several lineages exhibit marked source biases in this global genome collection, consistent with our local patterns in Pingguo. For instance, ST23 is a top human‐associated lineage, while ST37 is highly prevalent in nonhuman isolates. Despite this, six of the ten most abundant STs are shared across sources (e.g., ST11, ST15, ST258, ST307). Furthermore, the structure between human and nonhuman sources is geographically dependent and appears to be mediated by the dominant regional STs. For example, ST11 was the leading lineage in both sources in China and Europe subsets, whereas in the U.S. subset, the principal point of overlap is ST258.

In both the global dataset and nearly all regional subsets, human isolates consistently show higher mean virulence and AMR scores than nonhuman isolates (Figure 5b). AMR gene and class burdens, however, vary by geography: in the China subset (*n* = 15068), nonhuman isolates carry more AMR genes (mean Δ = 4.5) and classes (mean Δ = 1.0; both *p* < 0.001), consistent with the Pingguo findings; in the U.S. (*n* = 13912) and Europe (*n* = 14238) subsets, the pattern reverses, with higher AMR gene and class burdens in human isolates. This finding is further supported by a previously constructed *K. pneumoniae* genome collection (11259 total; curated nonredundant 9705) [26], which shows the same regional contrast: higher AMR burden in nonhuman isolates from the China subset (|Cliff's δ| = 0.459, medium effect) but in human isolates from the U.S. subset (|Cliff's δ| = 0.582, large effect). Among nonhuman reservoirs, overall AMR burden ranked China > Africa > Europe > the U.S. (pairwise tests under equal‐*n* resampling, Tukey's HSD, *p* < 0.001). Detailed dataset information and analytical results are provided in Data S8. Source‐resolved analyses further indicate that the elevated nonhuman AMR burden observed in the China subset is attributable largely to animal and environmental isolates (direct comparisons between China and the U.S. subsets, *p* < 0.001; Figure 5d), whereas food isolates show no comparably clear difference.

Density‐contour plots of AMR‐virulence trait (Figure 5c) based on our global database also show pronounced geographic contrasts. In the China and Africa subsets, human and nonhuman isolates exhibit high cross‐niche similarity in trait profiles. By contrast, the Europe and the U.S. subsets exhibit stratification, with nonhuman isolates concentrated in a low‐virulence, low‐AMR trait. A similar pattern is also observed for AMR score, with overlap between human and nonhuman isolates in the China and Africa subsets but clearer separation in the Europe and U.S. subsets (Figure S4a, Supporting File 2).

### 2.5 Lineage and Mobilome‐Associated Connectivity Contribute to Geographic AMR Divergence

**FIGURE 6:**
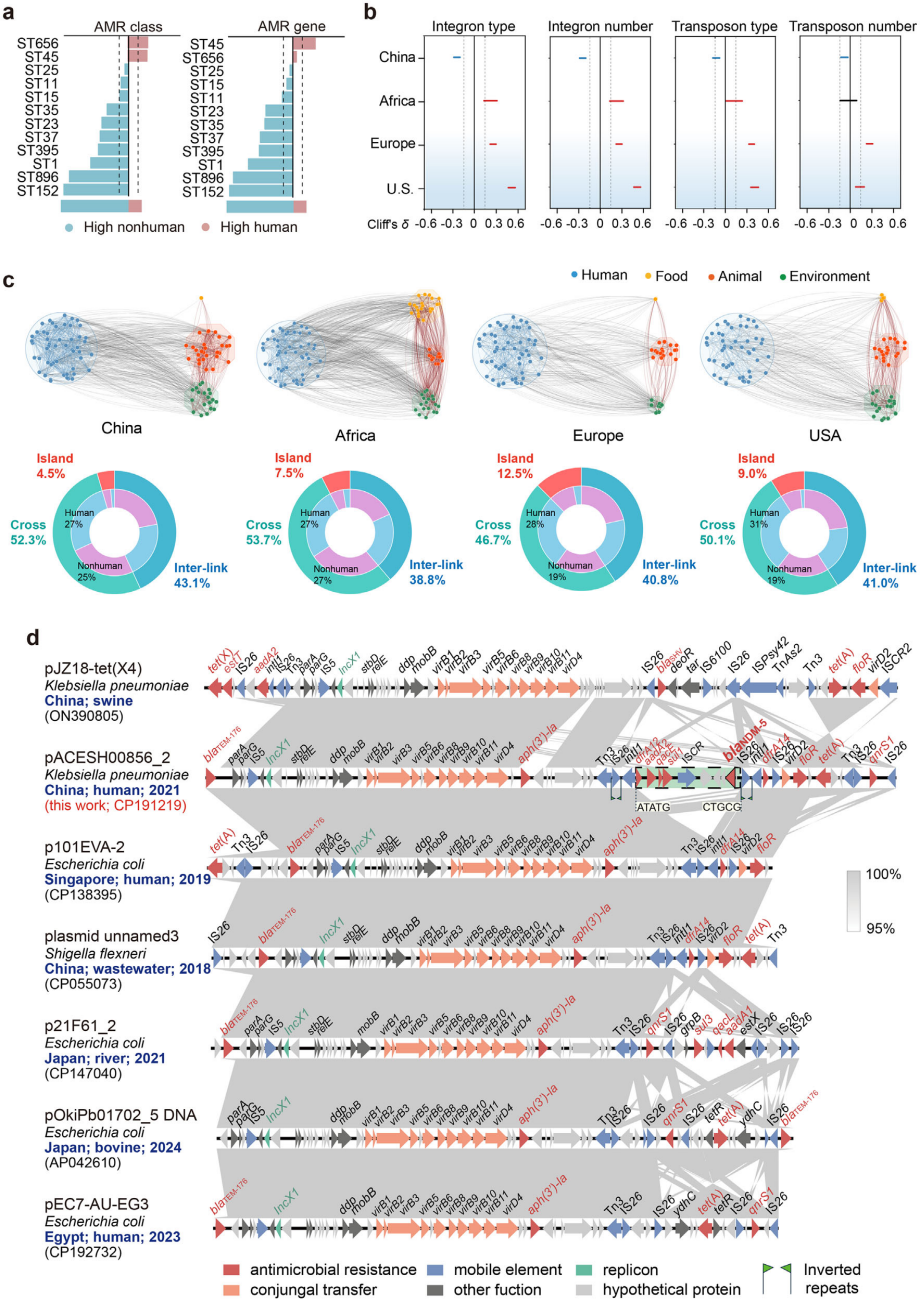
Eco‐geographic divergence in AMR burden, mobile genetic elements, and plasmid replicon‐sharing connectivity. (a) Within shared STs among Chinese isolates, effect sizes (Cliff's δ, human vs. nonhuman) for AMR classes and genes. Bars show the number of STs with δ > 0 (red, human>nonhuman) or δ < 0 (blue, human<nonhuman). Most STs skew blue, indicating that nonhuman sources carry higher AMR loads even after controlling for lineage. (b) Effect sizes (Cliff's δ, human vs. nonhuman) for integron and transposon metrics by region (types and counts). The vertical dashed line marks the small‐effect threshold |δ| = 0.147. China shows negative δ (nonhuman > human), whereas Africa/Europe/U.S. tend to show positive δ (human > nonhuman). (c) Regional plasmid‐exchange networks. Nodes are genomes colored by source (human, food, animal, environment). For visualization, equal‐size subsets are shown (human *n* = 100, nonhuman *n* = 100). We display the cross‐source components: all nodes that have at least one path linking different sources, and all edges among those nodes (islands and nodes confined to within‐source components are omitted). An edge links genomes sharing ≥3 plasmid replicon types. To balance sources, human genomes were down‐sampled to the nonhuman count and networks recomputed 100 times. The accompanying pie charts show mean proportions of island (no edges), inter‐link (nodes with ≥1 edge but outside the cross component, i.e., within‐source only), and cross (nodes in the cross‐source component). Cross‐source participation is highest in China and Africa, with substantial nonhuman involvement. (d) Sequences comparison of *bla*_NDM‐5_‐positive plasmids, including the IncX1 plasmid pACESH00856 and some other IncX1 plasmids from GenBank.

A linear mixed‐effects model indicated that bacterial lineage (represented by ST) accounts for 44.8% of variance in AMR gene burden across regions and sources. In the China subset, predominant STs among nonhuman isolates exhibit higher AMR gene burdens than predominant STs among human isolates (Figure S4c, Supporting File 2). Yet, over half of the burden variation remained unexplained, suggesting additional genomic drivers. To control for lineage, we compared AMR burdens among isolates belonging to the same STs across different niches, including only STs with at least 10 isolates per source. In the China subset, nonhuman isolates consistently carry significantly greater AMR gene burdens than their human isolates, even within the same ST (Figure 6a). This pattern is associated with enrichment of AMR genes conferring resistance to tigecycline, macrolides, and rifamycin in nonhuman reservoirs (Figure S4b, Supporting File 2).

We further found that the mobile genetic elements (MGEs) burden is closely associated with source‐specific AMR burden. Across the global dataset, AMR gene burdens are positively correlated with both integron type/number (e.g., Spearman's *ρ* = 0.69, *p* < 0.001) and transposon type/number (e.g., *ρ* = 0.38–0.51, *p* < 0.001). The distribution of these MGEs across ecological niches is also consistent with regional AMR patterns. In both the Pingguo survey (Table S1) and the broader China subset (Figure 6b), nonhuman isolates, particularly those from animals and the environment, carry a greater diversity and abundance of integrons and transposons than human isolates. Conversely, in the U.S. and Europe subsets, MGEs are more abundant in human isolates, aligning with their higher AMR burdens (Figure S5, Supporting File 2). This suggests that MGEs may be an important contributor to regional and source‐specific differences in AMR burden. Consistently, replicon‐sharing connectivity inferred from plasmid replicon similarity network, used here as a proxy for cross‐niche dissemination potential, shows marked regional differences. Human–nonhuman connectivity based on plasmid replicon similarity is significantly higher in the China and Africa subsets than in the U.S. and Europe subsets (Figure 6c). In the China and Africa subsets, nonhuman isolates also occupy more central positions in these cross‐niche connectivity networks, whereas in the U.S. and Europe subsets they are much less prominently represented.

To further investigate plasmid sharing patterns, we performed a comprehensive clustering analysis on reconstructed plasmids from the Pingguo isolates. Among the 917 strains analyzed, plasmids are identified in 851 isolates (93.8%), yielding a total of 2056 plasmids. These are partitioned into 89 plasmid clusters (PCs) and 112 singletons based on single‐linkage clustering. The resulting similarity network (Figure S6a, Supporting File 2) reveals a highly structured transmission landscape dominated by PC_1, a large interconnected component spanning 269 distinct STs (Figure S6b, Supporting File 2). Hospital isolates are frequently intermingled with farm and community isolates, while nodes representing human, animal, and environmental hosts form interconnected sub‐networks, consistent with extensive cross‐niche plasmid sharing. PC_1 is detected across 269 distinct STs, substantially exceeding the next most prevalent clusters (PC_21 and PC_3, each spanning 44 STs). In contrast, most PCs (*n* = 86) exhibit narrow host‐range distributions, spanning fewer than 5 STs, whereas singletons accounted for 74 unique ST‐specific plasmid types. Thus, a single dominant cluster (PC_1) accounts for 31.6% of all plasmid–ST associations, indicating a highly skewed host‐range hierarchy. Mobility typing further indicates that conjugative plasmids within PC_1 serve as primary vectors for horizontal gene transfer across different niches. Reciprocal analysis at the lineage level reveals marked variation in plasmid‐cluster diversity among STs (Figure S6c, Supporting File 2). ST15 and ST23 harbor 20 and 19 distinct PCs, respectively, whereas most STs carry limited PC diversity (≤3 types). Notably, a small subset of epidemic lineages, despite representing a minority of all strains, collectively harbor most of the total PC diversity. This suggests that cross‐niche plasmid sharing in the local system may be disproportionately maintained by a limited number of successful lineages and specific plasmid groups.

Complete genome assemblies for several Pingguo isolates provided genomic evidence consistent with MGEs mediating AMR gene dissemination across clonal and ecological boundaries. For example, the carbapenemase gene *bla*_NDM‐5_ was detected in three phylogenetically distinct hospital isolates in Pingguo: two on IncX3 plasmids and one on IncX1 plasmid. To our knowledge, this is the first report of *bla*_NDM‐5_ on an IncX1 plasmid in *K. pneumoniae*. Although this IncX1 variant has not been widely linked to carbapenem resistance, its conserved backbone is already broadly distributed across regions, sources, and bacterial hosts (Figure 6d), indicating a latent risk of future *bla*_NDM‐5_ acquisition and spread. Within the IncX3 group, the high‐virulence isolate ACESH02857 (virulence score = 3, ST458) carries a 46‐kb plasmid, whereas the low‐virulence isolate ACESH02121 (virulence score = 0, ST7103) carries a larger 49‐kb variant containing an additional IS*Kra4*‐like element (IS*Kpn19* family transposase) (Figure S7, Supporting File 2). Similar *bla*_NDM‐5_ cassettes flanked by related MGEs are found in *K. pneumoniae* isolates from different sources and in non‐*K. pneumoniae* pathogens, suggesting potential cross‐source and cross‐species dissemination of *bla*_NDM‐5_. Further evidence comes from colistin resistance gene *mcr‐8.2*. It is found in a highly virulent ST65 isolate (virulence score = 5), within a ∼100‐kb genomic region, and in a low‐virulence ST1‐1LV/ST6924 isolate (virulence score = 0), on a ∼37‐kb plasmid. In both cases, this gene is embedded within a highly conserved unit framed by identical insertion sequences. The same structure has been identified previously on plasmids from chicken and sewage samples (Figure S8, Supporting File 2). Together, these findings support an MGEs‐driven mechanism underlying the cross‐niche dissemination of AMR genes.

### 2.6 Temporal Dynamics of Virulence‐Resistance Convergence

**FIGURE 7:**
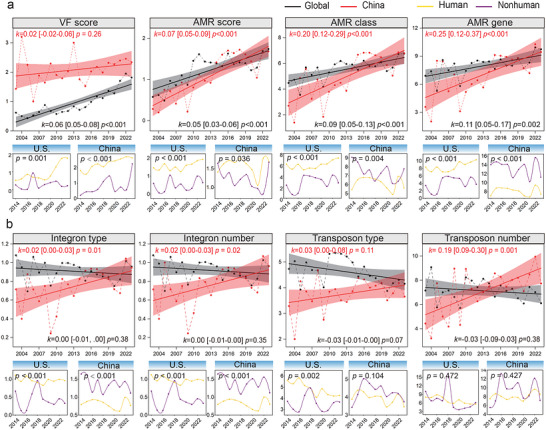
Temporal dynamics of virulence (VF) scores, antimicrobial resistance (AMR) profiles, and mobile genetic elements (MGEs) in *K. pneumoniae* genomes. (a) Trends in virulence and AMR. Mean VF score shows a significant global increase but no significant trend in China; the mean AMR score, AMR classes, and AMR genes increase in both datasets (see *R^2^* and *p*). Human isolates have higher mean VF and AMR scores in both U.S. and China; mean AMR gene diversity and counts are higher in humans in the U.S. but higher in nonhuman isolates in China. (b) Trends in typical MGEs including integrons and transposons. China shows significant increases in integron types, integron counts and transposon counts, whereas global trends are flat; transposon‐type richness shows no significant trend in either set. Integron metrics significantly differ by source in both countries. Upper panels: Annual trends (yearly means). Solid lines show ordinary least‐squares fits with 95% confidence intervals (Cis); shaded areas are confidence bands. *k* denotes the annual growth rate (regression slope, units per year; reported as estimate with 95% CI). Lower panels: Source‐stratified time series for the U.S. and China (yellow = human, purple = nonhuman). *p*‐values are from Mann–Whitney U tests (human vs. nonhuman).

Temporal analysis reveals a trend toward convergence of virulence and AMR burdens. Over the past two decades, the global mean virulence score of *K. pneumoniae* has steadily increased (annual growth rate 0.06 yr^−^^1^), approaching the high baseline observed in the China subset (Figure 7a). In parallel, global mean AMR gene and class burdens have also increased since 2003, consistent with rising antibiotic consumption [28] and overall AMR prevalence [29]. We identified significant temporal correlations between annual global antibiotic use [28] and *K. pneumoniae* AMR gene burdens and AMR scores (Spearman's *ρ* = 0.762 and 0.714, *p* = 0.028 and 0.046) (Data S9). Notably, annual growth rates for AMR gene and class burdens in the China subset (0.25 and 0.20 yr^−^^1^, respectively) are approximately double the corresponding global rates (0.11 and 0.09 yr^−^^1^, respectively). Consequently, China's mean AMR gene/class burden, initially below the global average, surpassed the global average around 2015. In the China subset, integron‐related metrics are also increased over time, paralleling the rise in AMR burden, whereas transposon‐related trends are weaker or less consistent (Figure 7b). Further stratification within regions shows that integron‐related metrics are significantly enriched in nonhuman isolates in the China subset, whereas they are significantly enriched in human isolates in the U.S. subset.

## 3 Discussion

Our findings challenge the assumption that dominant AMR reservoirs are globally uniform. Instead, we demonstrate that AMR reservoir dominance in *K. pneumoniae* is eco‐geographically structured, shaped by the interplay among regional lineage composition, mobilome amplification, and cross‐niche connectivity. In high‐connectivity settings, nonhuman isolates, particularly those from animals and the environment, harbor higher burdens of multidrug resistance platforms and are enriched in integrons. In more segregated contexts, AMR burden remains concentrated within human‐associated lineages, with comparatively limited cross‐niche amplification. This framework helps explain why prior One Health studies have reached differing conclusions about the relative importance of human and nonhuman reservoirs [30, 31, 32, 33]. These contrasts may reflect distinct ecological settings rather than methodological inconsistency. Crucially, overall AMR burden and clinically high‐risk determinants can also be ecologically decoupled. Nonhuman reservoirs may preferentially support broad resistance load and multidrugresistance platforms, whereas human isolates are more strongly enriched for clinically high‐risk determinants, including carbapenem resistance, colistin resistance, and hypervirulence. By incorporating eco‐geographic structure, our analysis shifts the central question from “which reservoir dominates?” to “under what ecological conditions does dominance arise, and for which dimension of risk?”

Geography is a structuring force in pathogen ecology and AMR dynamics [34, 35, 36], and our results are consistent with that principle. In *K. pneumoniae*, both AMR reservoir dominance and the degree of human‐to‐nonhuman coupling vary across regions. Compared with the U.S. and Europe subsets, the China and Africa subsets exhibit higher AMR burdens in nonhuman reservoirs, stronger cross‐niche connectivity, and greater network centrality of nonhuman isolates. These regional differences can be explained in part by the geographically structured lineage composition of *K. pneumoniae* [3, 26, 37, 38], as regional shifts in dominant STs generate baseline differences in AMR burden. Meanwhile, higher AMR burdens also coincide with higher MGE loads, supporting an additional role for mobilome‐mediated amplification in shaping regional AMR accumulation and redistribution [24, 39, 40, 41, 42]. Consistent with this, the China subset appears to follow a faster AMR trajectory than the global background and shows stronger integron‐enriched signals in nonhuman reservoirs, potentially reflecting greater anthropogenic pollution pressure [43]. In such high‐connectivity settings, nonhuman reservoirs may function not only as sites of AMR accumulation but also as intermediates that acquire resistance determinants from the broader environmental resistome and facilitate their subsequent dissemination into human‐associated *K. pneumoniae* lineages [44, 45, 46].

Supportive evidence from the Pingguo assemblies further refines this interpretation. Placed in the context of prior One Health cross‐sector studies, the Pingguo survey both confirms and extends existing patterns [12, 13, 25, 27]. As in earlier reports, in Pingguo, clone sharing between clinical and nonclinical settings is limited and ecological structuring remains strong. Clinically significant determinants, including carbapenemase genes and hypervirulence loci, are still concentrated in human clinical isolates, with only sporadic occurrences outside the hospital and comparatively limited geographic variability. Yet overall AMR gene composition in Pingguo shows much weaker human‐to‐nonhuman separation than clonal backgrounds do (Figure S9, Supporting File 2), suggesting multidrug resistance may disseminate more readily than clones. MOB‐suite analysis strengthens this interpretation by revealing a highly structured plasmid‐cluster landscape in which a small number of widely distributed plasmid clusters connect multiple STs and ecological compartments. This finding suggests that cross‐niche connectivity is not diffuse but depends disproportionately on a limited set of successful lineage–plasmid combinations. By extension, regional differences in the distribution of these lineage and plasmid backgrounds may help explain why human–nonhuman coupling varies across settings and why dominant AMR reservoirs differ between regions. This supports a model in which lineage structure sets the regional background for AMR accumulation, whereas mobilome‐associated exchange modulates the extent of cross‐niche amplification. Community sampling in Pingguo further revealed active human–household environmental links, highlighting an interface often under‐sampled in prior works [12, 25]. More broadly, such pattern may reflect regional differences in exposure and contact structure, for example, more frequent human‐animal contact in resource‐limited settings, which potentially facilitates the dissemination of resistance determinants [47].

Overall, these observations support a tiered, region‐tailored One Health surveillance framework grounded in measurable ecological indicators. In this framework, integron load and cross‐niche connectivity provide practical indicators of regional exchange capacity, as both can be derived from routine whole‐genome sequencing data and scaled across surveillance systems without requiring plasmid‐resolved assemblies. In high‐connectivity settings, surveillance should extend beyond high‐risk clinical determinants to include agricultural and environmental reservoirs, where multidrugresistance platforms may expand upstream. In lower‐connectivity settings, clinical surveillance can remain the primary anchor, with mobilome‐informed metrics serving as sentinels for emerging spillover. Together, these findings argue for calibrating sampling intensity and compartmental weighting to local AMR ecology.

Despite these insights, several limitations remain. The Pingguo collection was conducted within a relatively narrow sampling window and should therefore be interpreted as a contemporaneous local snapshot. As such, it may not fully capture temporal variation in AMR burden and reservoir distribution. In addition, the inherent biases of public datasets, including uneven sampling strategies, sequencing technologies, metadata quality, and relatively limited and fragmented African data, limit their representativeness for national‐level inference, although they still provide useful comparative signals at regional and ecological scales. The directional concordance between the Pingguo survey and the China subset of the public genome collection supports the same overall pattern but does not justify direct extrapolation from one locality to China as a whole. Therefore, more longitudinal, multi‐centric, and multi‐season surveys across diverse ecological compartments and regions will be important to determine the temporal stability and broader generalizability of the observed patterns. Future work should also quantify local selective pressures, including agricultural antibiotic use and environmental contamination, that may shape AMR in nonhuman reservoirs, and investigate mechanisms of niche separation in high‐income regions.

In summary, AMR reservoir dominance in *K. pneumoniae* is not universal but depends on regional ecological context. Differences in lineage composition, mobilome amplification, and cross‐niche connectivity shape distinct exchange regimes across regions, with direct consequences for where resistance accumulates and how it should be monitored. These results support a precision One Health framework in which local AMR ecology is first diagnosed and surveillance priorities are then tailored accordingly, rather than inferred from a single global template.

## 4 Experimental Section

### 4.1 Study Design and Sampling Overview

We conducted a cross‐sector genomic survey of *K. pneumoniae* in Pingguo (23.32 N, 107.58 E), Guangxi, a county‐level city in southern China. Pingguo provides a suitable setting because healthcare, livestock production, food consumption, and environmental interfaces are closely connected. The study area encompassed sampling sites within a 30‐km radius: one hospital, two villages, three livestock farms, one agricultural market, and one slaughterhouse (Figure 1a). Villages were selected to maximize ecological representativeness and local connectivity, including proximity to livestock production, markets/slaughtering facilities, access to natural water bodies, and high household self‐sufficiency.

From November 2020 to November 2021, monthly sampling at the hospital's microbiological diagnostic laboratory yielded 4247 non‐duplicate clinical isolates from patients with suspected infections (Data S1). 748 clinical samples were positive for *K. pneumoniae*, yielding 750 isolates, with collection sources spanning multiple departments and a range of clinical specimen types (Data S2).

In June 2021, we conducted an intensive one‐month cross‐sector sampling campaign across community and farm settings. Human participants were primarily recruited from the two selected villages through household‐based enrolment with informed consent. Additional samples included livestock manure, farm worker feces, household surface swabs, market foods, and environmental matrices (soil, water, and plants), enabling linkage analyses across human, animal, food, and environmental compartments (Figure 1a). Detailed sampling procedures and sample details are provided in Text S1 (Supporting File 1). While we attempted to minimize bias, residual selection and sampling biases are possible and are discussed as limitations.

### 4.2 Strain Isolation, Identification, Genome Sequencing, and Bioinformatic Analysis

All samples were transported on ice and processed within 24 h. *K. pneumoniae* isolates were recovered using source‐specific culture workflows, including direct plating for stool samples and enrichment‐based isolation for environmental, food, and household swab samples. Identification of bacterial species was performed using MALDI‐TOF MS. Strains confirmed as *K. pneumoniae* by MALDI‐TOF MS were preserved at −80°C. In total, 917 unique *K. pneumoniae* isolates were recovered from 5384 samples (Sampling/workflow summaries in Data S1 and S2 and Figure 1b).

Antimicrobial susceptibility testing was performed for all 917 isolates using *Escherichia coli* ATCC 25922 and *K. pneumoniae* ATCC 700603 as quality control strains. A complete list of antimicrobials tested, isolate‐level minimum inhibitory concentration values, and categorical susceptibility results (0 = susceptible; 1 = resistant) is provided in Data S3.

Genomic DNA from 917 isolates was subjected to Illumina whole‐genome sequencing (2 × 150 bp). Raw reads and/or genome assemblies have been deposited in NCBI, and the corresponding accession numbers are provided in Data S2. For four isolates carrying specific AMR genes (ACESH00856, ACESH02121, ACESH02857, and ACESH00926), long‐read Nanopore sequencing was additionally performed to generate complete assemblies for plasmid‐resolved analyses. Detailed protocols are provided in Text S2 (Supporting File 1).

Because MALDI‐TOF MS cannot distinguish members of the KpSC [3], species assignments were refined using whole‐genome sequence‐based classification. After excluding non‐*K. pneumoniae* KpSC members, 820 *K. pneumoniae* isolates were retained for core‐genome SNP phylogenetic analysis. We reconstructed a core‐genome SNP phylogeny for the 820 confirmed isolates and annotated the tree with lineage, AMR, virulence, and sampling metadata to examine phylogenetic structuring across niches. As a complementary analysis, we also generated a rapid whole‐genome distance‐based overview for all 917 initially recovered isolates using Mash‐derived pairwise distances. Software, parameters, and phylogenetic procedures are provided in Text S2 (Supporting File 1). To infer putative transmission patterns, we used pairwise SNP distances derived from the recombination‐filtered core‐genome alignment to construct SNP‐threshold networks [12] and compare clustering dynamics. This matrix, detailing the SNP distances between all isolate pairs, is provided in Data S4. Because the hospital and community datasets differed substantially in size, we combined a simple sharing index with size‐controlled iterative subsampling [48] to enable normalized comparisons of sharing‐linked isolates and event counts across settings. Community isolates were further partitioned into intra‐household and inter‐household clonal sharing groups for comparative analyses. SNP thresholds, resampling procedures, and statistical tests are provided in Text S2 (Supporting File 1).

All 917 isolates were characterized using Kleborate, while Kleborate‐derived AMR and virulence metrics were used for clade‐level and cross‐niche comparisons (complete output in Data S2). MGEs, including integrons and transposons, were predicted for *K. pneumoniae* genomes using the BacAnt platform [49]. MGEs data for 820 *K. pneumoniae* isolates were shown in Data S5 and used for downstream ecological and comparative analyses. Plasmid sharing network analysis was performed based on plasmid reconstruction and characterization using MOB‐suite. For comparative analyses, isolates derived from animal manure, household swabs, food, soil, water, and plants were aggregated into a single nonhuman category. Detailed scoring criteria, databases, and post‐processing steps are provided in Text S2 (Supporting File 1).

### 4.3 Global *K. pneumoniae* Genome Data Collection and Analysis

A final dataset of 69184 public *K. pneumoniae* genomes, retrieved from GenBank Assembly as of August 8, 2024, was used for global comparative analyses. These genomes were derived from 69250 curated KpSC assemblies after de‐duplication and taxonomic validation (Figure S10, Supporting File 2, and comprised 2798 complete genomes, 295 chromosome‐level assemblies, 56991 contigs, and 9100 scaffolds. Virulence and AMR determinants were annotated using Kleborate, and MGEs using BacAnt. The accession list and summary results are provided in Data S6. Temporal trends were assessed by linear regression of virulence score, AMR score, AMR gene number, and AMR class number against isolation year.

To compare virulence, AMR, and MGEs metrics across geographic regions (global, China, Africa, Europe, and the U.S.) while controlling for sample size disparities, a bootstrap resampling approach was employed. The dataset was first stratified by source (human versus nonhuman). The nonhuman compartment comprises all isolates derived from animal‐, environmental‐, and food‐associated sources, as annotated in the original metadata. For each source type, we performed 1000 bootstrap iterations. In each iteration, a fixed number of isolates were randomly sampled with replacement, and the mean values of virulence, AMR, and MGEs metrics were calculated. Resampling was performed at multiple sizes (e.g., 50, 100, 150 for nonhuman; 500, 1000, 1500 for human) to ensure the robustness of the findings across different sampling depths, guided by the smallest sample size in each source category. The resulting distributions of mean values (*n* = 1000 per group) were then statistically compared using one‐way ANOVA followed by Tukey's HSD post‐hoc test. To quantify the magnitude of any significant differences across sources, Cliff's δ was calculated as a non‐parametric measure of effect size.

To investigate AMR differences between human and nonhuman sources for shared genetic lineages, we conducted a source‐specific analysis stratified by ST. Only STs with a prevalence of at least ten isolates in both the human and nonhuman categories were included. For each of these retained STs, the AMR score and AMR burdens were directly compared between human‐ and nonhuman‐derived isolates using the Mann–Whitney U test. To quantify the magnitude of any significant differences, Cliff's δ was calculated as a non‐parametric measure of effect size, with values closer to 1 or ‐1 indicating greater effects.

Meanwhile, all genomes underwent initial quality control following the criteria established by Lam et al. [26], and low‐quality assemblies were excluded. For each geographic region (China, the U.S., Europe, Africa), a non‐redundant genome set was then generated by clustering the remaining high‐quality genomes with a pairwise Mash distance ≤ 0.0003 and selecting one random representative per cluster. This resulted in 5824, 6717, 9185, and 1305 non‐redundant, high‐quality genomes for China, the U.S., Europe, and Africa, respectively, and the corresponding accession numbers for these genomes are listed in Data S7. Plasmid replicon details for these high‐quality genomes were profiled using Abricate with the PlasmidFinder database (v4.7.2). For each region, a plasmid replicon similarity network was constructed where nodes represent genomes, and an edge indicates the sharing of plasmid replicon types. To address source imbalance, we down‐sampled human genomes to the nonhuman sample size and recomputed networks 100 times, summarizing (i) the proportion of isolated nodes, (ii) the fraction of within‐ vs. between‐source nodes, and (iii) assortative by source across iterations; equal‐size subsets (human *n* = 100, nonhuman *n* = 100) were used for visualization.

Additional statistical analysis and plotting can be found in Text S3 (Supporting File 1). It is important to note that AMR gene burden was defined as the number of acquired AMR genes detected per isolate from the Kleborate output. AMR class burden was defined as the number of antimicrobial classes represented by these genes per isolate. Unless otherwise specified, AMR burden refers to these per‐isolate genome‐level metrics, rather than AMR gene abundance. Moreover, AMR burdens are distinguished from the Kleborate AMR score, which only summarizes clinically important resistance determinants (extended‐spectrum β‐lactamases, carbapenemase, or colistin resistance genes) rather than overall AMR gene load.

## Ethics Statement

This research received ethical approval from the Ethics Committee of the First Affiliated Hospital of Zhejiang University, with Ethics Approval Number: (2021) IIT Quick Review No. 631. Informed consent was obtained from all participants prior to sample collection.

## Conflicts of Interest

The authors declare no conflicts of interest.

## Data and Code Availability

All data are available in the main text and the Supplementary Materials. Raw reads and/or genome assemblies for the 917 isolates collected in this study have been deposited in NCBI, and the corresponding accession numbers are provided in Data S2. The full accession list for the 69184 public *K. pneumoniae* genomes retrieved is provided in Data S6. The complete Data S6 table, including full results from the Kleborate and MGEs analyses, has been deposited in Zenodo (https://doi.org/10.5281/zenodo.19536827). Accession numbers for the non‐redundant, high‐quality regional subsets further filtered from Data S6 are provided in Data S7. Custom scripts for public genome retrieval and preprocessing, plasmid replicon similarity network analysis, clonal sharing event analysis, linear mixed‐effects modeling, statistical analyses, and resampling are available at the GitHub repository [https://github.com/HuiLinZAAS/klebsiella‐amr‐ecogeography‐mobilome‐connectivity].

## Supporting information




**Supporting File 1**: advs75537‐sup‐0001‐SuppMat.docx.

**Supporting File 2**: advs75537‐sup‐0002‐SuppMat2.docx.

**Supporting File 3**: advs75537‐sup‐0003‐DataS1.xlsx.

**Supporting File 4**: advs75537‐sup‐0004‐DataS2.xlsx.

**Supporting File 5**: advs75537‐sup‐0005‐DataS3.xlsx.

**Supporting File 6**: advs75537‐sup‐0006‐DataS4.xlsx.

**Supporting File 7**: advs75537‐sup‐0007‐DataS5.xlsx.

**Supporting File 8**: advs75537‐sup‐0008‐DataS6.xlsx.

**Supporting File 9**: advs75537‐sup‐0009‐DataS7.xlsx.

**Supporting File 10**: advs75537‐sup‐0010‐DataS8.xlsx.

**Supporting File 11**: advs75537‐sup‐0011‐DataS9.xlsx.

**Supporting File 12**: advs75537‐sup‐0012‐DataS10.xlsx.
