# Supplementary material for: Eco‐Geography Reverses Dominant AMR Reservoirs in Klebsiella pneumoniae: Integron‐Rich Mobilomes and Cross‐Niche Connectivity
Source: Adv Sci (Weinh). 2026 May 6;13(42):e75537. doi: 10.1002/advs.75537 (PMC13336099; doi:10.1002/advs.75537)
Supplement: Supplementary file 1 — Supporting File 1: advs75537‐sup‐0001‐SuppMat.docx. [file ADVS-13-e75537-s004.docx]

**Supplementary Methods**

**Eco-geography reverses dominant AMR reservoirs in *Klebsiella pneumoniae:* integron-rich mobilomes and cross-niche connectivity**

Hui Lin,^1^† Biao Tang,^2^† Hao Xu,^3^† Jiajie Qian,^4^† Feifan Shi,^1,5^ Junhui Zhao,^6^ Xiaorui Mao,^2^ Xiaohe Hu,^2^ Ruishan Liu,^3^ Wenhong Liu,^7^ Xiawei Jiang,^7^* Beiwen Zheng,^3,8,9^* Guoping Zhao ^2,10^

^1^ State Key Laboratory for Quality and Safety of Agro-Product, Zhejiang Provincial Key Laboratory of Agricultural Microbiomics, Institute of Environment, Resource, Soil and Fertilizer, Zhejiang Academy of Agricultural Sciences, Hangzhou, PR China

^2^ Key Laboratory of Systems Health Science of Zhejiang Province, School of Life Science, Hangzhou Institute for Advanced Study, University of Chinese Academy of Sciences, Hangzhou, PR China

^3^ State Key Laboratory for Diagnosis and Treatment of Infectious Diseases, National Clinical Research Center for Infectious Diseases, National Medical Center for Infectious Diseases, Collaborative Innovation Center for Diagnosis and Treatment of Infectious Diseases, The First Affiliated Hospital, Zhejiang University, Hangzhou, PR China

^4^ Department of Gastrointestinal Surgery, The First Affiliated Hospital, College of Medicine, Zhejiang University, Hangzhou, PR China

^5^ Xianghu Laboratory, Hangzhou, PR China

^6^ Department of Clinical Laboratory, The Second Affiliated Hospital of Wannan Medical College, Wuhu, PR China

^7^ School of Basic Medical Sciences, Zhejiang Chinese Medical University, Hangzhou, PR China

^8^ Yuhang Institute of Medical Science Innovation and Transformation, Hangzhou, PR China

^9^ Jinan Microecological Biomedicine Shandong Laboratory, Jinan, PR China

^10^ Department of Microbiology, School of Life Sciences, Fudan University, Shanghai, PR China

† Contributed equally: Hui Lin, Biao Tang, Hao Xu, and Jiajie Qian.

***** Corresponding authors: Beiwen Zheng and Xiawei Jiang.

E-mail: zhengbw@zju.edu.cn; xiaweijiang@zcmu.edu.cn.

**This PDF file includes: Text S1 to S3**

**1. Text S1 Study design, site selection, and sampling procedures**

***Study design.*** We conducted a large cross-sector genomic survey of *K. pneumoniae* in Pingguo. This region was chosen due to its suitability to capture cross-niche transmission within a self-contained ecosystem and its integrated urban, agricultural, and community environments. Specifically, (i) the local secondary hospital provides primary care to nearby rural villages; (ii) livestock production and food consumption are closely integrated within the community; and (iii) pandemic-related travel restrictions during 2020-2021 minimized external microbial introductions, offering a unique natural context to examine local transmission dynamics.

***Site selection.*** To ensure ecological representativeness and internal connectivity, largely self-sufficient villages were selected based on the following criteria: (i) proximity to livestock farms (pig or poultry), agricultural markets, and, where possible, slaughtering facilities; (ii) availability of natural water sources, such as rivers, ponds, or irrigation canals; and (iii) a high degree of household self-sufficiency, with residents relying mainly on homegrown crops and animal husbandry. Based on these criteria, Dao’e and Xiancao villages were selected for community-based sample collection. The study area encompassed sampling sites within a 30-km radius: one hospital, two villages, three livestock farms, one agricultural market, and one slaughterhouse.

***Hospital-based sampling (November 2020 to November 2021).*** Monthly sampling at the hospital's microbiological diagnostic laboratory yielded 4,247 non-duplicate clinical isolates from patients with suspected infections. 748 clinical samples were positive for *K. pneumoniae*, yielding 750 isolates, with collection sources spanning multiple departments and a range of clinical specimen types.

***Intensive cross-setting sampling (June 2021).*** In June 2021, we undertook a one-month, intensive sampling efforts across diverse sources in Pingguo. Participants were mainly recruited from two selected villages. Initially, a total of 100 households from these villages were randomly invited to participate. After obtaining informed consent, household members were requested to provide faecal samples and complete questionnaires detailing demographic characteristics (age, gender, occupation, etc.), socioeconomic status (household income, educational attainment, etc.), and knowledge of antibiotic usage. Ultimately, samples were obtained from 98 households, including 44 households (118 residents) distributed across 14 hamlets in Dao'e Village and 43 households (95 residents) across 4 hamlets in Xiancao Village. In parallel, three nearby livestock farms (two poultry farms and one pig farm) were selected for animal manure sampling. Workers at these farms were also invited to provide fecal samples, resulting in 7 samples from poultry farm workers. Animal feces were collected from pigs and chickens at both household and commercial farms. Household swabs included bathrooms, door handles, and furniture. Food samples consisted of vegetables, pork, and chicken from agricultural market. Environmental samples included soils, waters, and plants. Sampling locations and numbers were selected to maximize ecological diversity and linkage across human, animal, and environmental niches. No substantial sources of bias likely to significantly impact the validity or generalizability of the results were identified.

**2. Text S2 Strain isolation, identification, sequencing, and bioinformatic analysis**

***Strain isolation and identification***

All samples were transported on ice to the laboratory and processed within 24 hours of collection. For stool samples (human or animal)*,* one gram was suspended in 9 mL sterile 0.85% saline solution (Sanggo Biotech, Shanghai, China) to achieve a 100-fold dilution. 10 μL of the diluted solution was evenly spread onto Columbia CNA blood-agar plate (bioMérieux, Shanghai, China) and incubated at 37°C for 18-24 hours. For environmental and food samples (meat), household swabs, soil, water, fresh plants, wastes, and vegetable food were firstly enriched in Luria-Bertani broth (Sanggo Biotech, Shanghai, China) via overnight incubation at 37 °C (≈ 16 h, 180 rpm). Cultures were then diluted 1: 100 in sterile 0.85% saline solution. 10 µL of each dilution were spread on MacConkey (MAC) agar plates (Oxoid, Basingstoke, UK) and incubated at 37 °C for 18-24 h. Colonies differing in morphology or colour were selected from all above-mentioned plates, and repeatedly sub-cultured on fresh Mueller–Hinton agar (MHA) plates (Oxoid, Basingstoke, UK) at 37 °C until single, morphologically uniform colonies were obtained. Meanwhile, hospital isolates stored at -80 °C were revived by streaking a loopful on MHA plates and incubating overnight at 37 °C. A single colony from each plate was then sub-cultured once on a fresh MHA plate under identical conditions. Identification of bacterial species was performed using matrix-assisted laser desorption ionisation-time of flight (MALDI-TOF MS; Bruker Daltonics, Bremen, Germany). Strains confirmed as *K. pneumoniae* by MALDI-TOF MS were preserved in 20% glycerol tubes at -80°C for future use. Of the 5,384 samples collected, 917 unique *K. pneumoniae* isolates were successfully cultured and processed for subsequent analysis.

***Antimicrobial susceptibility testing***

Minimal inhibitory concentrations (MICs) were determined on Mueller-Hinton media following standard protocols for all 917 local *Klebsiella* sp. isolates. Broth microdilution was employed for polymyxin B and tigecycline, while agar dilution was used for all other antimicrobials tested. Susceptibility was interpreted using clinical breakpoints from the Clinical and Laboratory Standards Institute (CLSI document M100-S30; https://clsi.org), with the exception of tigecycline, for which U.S. Food and Drug Administration (FDA) criteria (https://www.fda.gov/drugs/development-resources/tigecycline-injection-products) were applied. *Escherichia coli* ATCC 25922 and *K. pneumoniae* ATCC 700603 served as quality control strains for all assays.

***DNA extraction and Illumina sequencing***

All isolates that are identified as *K. pneumoniae* via MALDI-TOF MS were subjected to genomic DNA extraction using the Gentra Puregene Yeast/Bact Kit (QIAGEN), according to the manufacturer’s protocol. DNA quality and quantity were assessed using a NanoDrop 2000 spectrophotometer (Thermo Fisher Scientific) and a Qubit 4.0 Fluorometer (Invitrogen). DNA integrity was confirmed by 1% agarose gel electrophoresis. Nextera XT DNA library construction kit (Illumina; cat. no. FC-131-1024) was applied for constructing genomic DNA library according to the manufacturer’s instructions. Libraries were subsequently sequenced on an Illumina NovaSeq 6000 platform (Illumina, San Diego, USA) to generate 2×150 bp paired-end reads. The resulting clean reads were then assembled de novo using SPAdes (v3.10.0) with default parameters.

***Nanopore sequencing and plasmid analysis***

*K. pneumoniae* strains carrying specific ARGs (strains ACESH00856, ACESH02121, ACESH02857, and ACESH00926) were sequenced on the Oxford Nanopore MinION platform (Nanopore Technologies, Oxford, United Kingdom). De novo assembly was performed for each isolate using Unicycler (v0.5.0), which integrates both the Nanopore long reads and the previously generated Illumina short reads. The resulting assemblies were then polished with Pilon (v1.24) to generate complete, circularized chromosome and plasmid sequences. The final assembly quality was assessed with QUAST (v5.3.0) to confirm completeness and contiguity. AMR genes were annotated on the final assemblies using ABRicate (v1.0.1; https://github.com/tseemann/abricate). For detailed plasmid analysis, ResFinder (v4.7.2; http://genepi.food.dtu.dk/resfinder) was used for AMR gene confirmation, and BLASTn was used to compare genetic contexts. Linear comparative genomic maps were generated with Easyfig (v2.2.3) and circular plasmid comparison plots were generated via BRIG (v0.95).

***Species assignment***

Given the difficulty in distinguish members of the *K. pneumoniae* species complexes (KpSCs) by MALDI-TOF alone (Wyres et al., 2020), we first applied the Pathogenwatch platform (https://pathogen.watch/) for species assignment of all isolates according to its full genomic sequence. By excluding other KpSC members, we obtained 820 *K. pneumoniae* isolates for subsequent phylogenetic analysis.

***Core-genome SNP-based phylogeny***

A maximum-likelihood phylogenetic tree was constructed from core-genome alignment was generated using Snippy (v4.6.0, https://github.com/tseemann/snippy) with a representative ST11 genome (GCA_000240185.2, hospital patient sputum, USA) as the reference. The initial alignment was cleaned of non-core regions using snippy-clean_full_aln. Subsequently, Gubbins (v3.3.5) was used to identify and remove regions of recombination, yielding a filtered alignment of polymorphic sites. SNP-sites (v2.5.1) was then applied to extract a final SNP alignment (225 bp in length). Using this alignment, a maximum-likelihood tree was inferred with IQ-TREE (v2.3.3) under the TIM2e+ASC+R2 model (selected as the best-fit model) with 1,000 bootstrap replicates. The final tree was visualized in iTOL (v7) and rooted using the midpoint rooting function. To annotate the phylogeny, a comprehensive genomic feature for each isolate was generated using Kleborate (v3.1.0). We extracted lineage distribution (ST and K-locus) and key AMR and virulence traits. This information, together with sample collection detail (setting and source), was converted into a binary or categorical data matrix. The matrix was then uploaded to the iTOL and displayed as annotated heatmaps with the tips of the phylogenetic tree, allowing for visual correlation between phylogeny and phenotype-associated traits.

***Rapid genome-wide comparison***

As a complementary analysis, the overall genomic distances among all 917 initial isolates were estimated using Mash (v2.0) (Ondov et al., 2016). A neighhor-joining tree was constructed from the resulting pairwise Mash distances using RapidNJ (v2.3.2) to provide a broad overview of the genomic diversity within the full dataset. This tree was then visualized in iTOL (v7) and annotated with key genomic features derived from Kleborate (e.g., AMR/virulence scores) and sample metadata (e.g., source) to enable a visual assessment of major clusters. For subsequent quantitative analysis, isolates in this tree were grouped into five major clades. The characteristics of each clade were then summarized by calculating the mean Kleborate virulence score (AVS) and AMR score (AAS). Furthermore, the diversity of isolate sources within each clade was quantified using the Shannon diversity index (*H’* = -∑PᵢlnPᵢ, where *Pᵢ* is the proportion of isolates from a given source *i*).

***Clonal sharing events*** ***inferring for 820 local isolates***

The core SNP alignment, generated as described above for phylogenetic analysis, was used to compute a full pairwise SNP distance matrix with snp-dists (v0.7.0; https://github.com/tseemann/snp-dists) (Thorpe et al., 2022). To visualize putative transmission links, the distance matrix was imported into Gephi (v0.10.1) to generate undirected and unweighted networks. Edges connecting isolates were retained at four distinct SNP thresholds: ≤ 10, ≤ 20, ≤ 80, and ≤ 100 SNPs, representing potential sharing events.

To enable a fair comparison of sharing dynamics between the large hospital collection (*n* = 712) and the smaller community collection (*n* = 94), we employed two normalization strategies. First, a simple sharing index was calculated for each primary setting (hospital and community). This was defined as the proportion of isolates belonging to at least one transmission cluster (i.e., having a link of ≤10 SNPs) relative to the total number of isolates in that setting. Second, as a more robust comparison, we performed a size-controlled iterative subsampling analysis (Muloi et al., 2022). We performed these analyses at multiple size-matched subsets (*n* = 50, 60, 70, and 80) and repeated 100, 1,000, and 10,000 iterations. As the results were consistent and stable across different iteration counts, all subsequent reporting and visualizations are based on the 10,000 iteration sets.

The community collection was further stratified into isolates involved in inter-household and intra-household transmission events. We then generated distributions of potential transmission event counts for each setting (within-hospital, total community, intra-household, inter-household) by repeatedly sampling size-matched subsets. Finally, the statistical significance of the difference between these distributions (e.g., intra-hospital vs. total community; intra-hospital vs. inter-household) was assessed using the Mann-Whitney U test. The expected range of transmission events for each setting was defined by the 95% confidence intervals from the resampling distributions.

***Genomic typing and scoring with Kleborate***

All 917 *Klebsiella* sp. isolates were characterized using Kleborate (v3.1.0). This tool was employed to confirm species identity, assign multilocus sequence types (STs) and KL within KpSC, and generate a comprehensive profile of AMR and virulence genes. Viruence and AMR scores were calculated based on the presence of key genetic determinants. The virulence score (0-5) was assigned based on the presence of the yersiniabactin (*ybt*), colibactin (*clb*), and aerobactin (*iuc*) loci as follows: 0 = none; 1 = *ybt* only; 2 = *clb* (with or without *ybt*); 3 = *iuc* only; 4 = *iuc* + *ybt*; and 5 = *iuc* + *ybt* + clb. The AMR score (0-3) was calculated based on the presence of extended-spectrum β-lactamases (ESBL) or carbapenemase genes, and predicted colistin resistance: 0 = no ESBL or carbapenemase; 1 = ESBL without carbapenemase; 2 = carbapenemase without colistin resistance; and 3 = carbapenemase with colistin resistance.

***Prediction of mobile genetic elements (MGEs)***

MGEs, including integrons and transposons, were identified in *K. pneumoniae* genomes using the BacAnt platform (http://www.bacant.net/BacAnt/) (Hua et al., 2021). Specifically, the IntegronDB and TransposonDB modules were employed to screen each genome against databases of integron structures and transposon-associated elements, respectively, under default parameters. Following this identification, the MGEs load for each isolate was quantified. Specifically, we processed the BacAnt output to calculate two metrics: (i) the total number of distinct transposon and integron classes (a measure of diversity), and (ii) the total count of genes carried by these elements (a measure of burden).

***Analysis of plasmid sharing networks based on MOB-suite***

Plasmid reconstruction and characterization were performed using MOB-suite (v3.0.1). Contigs from hybrid assemblies were screened for plasmid-specific sequences using mob_recon, and plasmid mobility was classified using mob_typer into conjugative (self-transmissible), mobilizable (dependent on helper plasmids), or non-mobilizable categories. Replicon types were determined using the PlasmidFinder database integrated within MOB-suite. For network analysis, pairwise Mash distances (v2.3; k = 21, s = 10,000) were calculated between all plasmids. Pairs with Mash distance ≤ 0.05 were considered connected. Plasmid clusters (PCs) were defined using single-linkage clustering (connected components algorithm) based on these pairwise similarities. Network visualization was generated using Cytoscape (v3.10.4). Nodes were colored by isolation setting (hospital, farm, community) and shaped according to host category (human, animal, water, household swab, soil, food).

**3. Text S3 Statistical analysis and plotting**

All statistical analyses were conducted in R (v4.0.4) unless otherwise specified. Standard comparative tests included the Mann-Whitney U test, Wilcoxon signed-rank test, one-way ANOVA, and Pearson’s χ² test for categorical data. Effect sizes for non-parametric comparisons were calculated as Cliff's delta using the cliff.delta() function from the effsize R package. Permutation tests (Thorpe et al., 2022) were conducted by randomly permuting source categories and cluster labels (species or sequence type, depending on the dataset; Data S10) 10,000 times. Empirical two-tailed *p*-values were calculated and subsequently adjusted for multiple comparisons using the Benjamini-Hochberg method in R (p.adjust() function. To enable robust comparisons between groups of disparate sizes (e.g., human vs. nonhuman sources across different geographic regions), we employed a resampling-based approach implemented via custom Python (v3.10.12) scripts.

To quantify the relative contribution of bacterial lineage to AMR variation, we fitted linear mixed-effects models (LMMs) and compared them with ordinary linear models (LMs). For AMR gene, the following model was fitted: AMR gene ~ Source + Region + (1|ST), where ST was included as a random intercept to account for phylogenetic structure, and Source (human vs. nonhuman) and Region (China, Africa, the U.S., Europe,) were treated as fixed effects. The corresponding linear model without the random term (model0) was used for comparison: AMR gene~ Source + Region. Model fitting was conducted using the lme4 and lmerTest packages in R (v4.3). The proportion of variance explained by lineage was calculated as: Phylogeny variance proportion = σ²_ST / (σ²_ST +σ²_residual). Model performance was compared using log-likelihood differences (ΔlogLik = logLik1-logLik0) and Akaike Information Criterion (AIC). A substantial improvement in model fit after including the random effect (larger ΔlogLik, smaller AIC) indicated that phylogenetic structure significantly contributed to AMR variation.

To visually compare the distribution of AMR-virulence trait between human and nonhuman sources, we generated two-dimensional density contour plots. To control for sampling bias resulting from the larger number of human isolates, we first performed a size-controlled subsampling for each geographic region (China, Africa, the U.S., Europe). The resampling size for each pairwise comparison was standardized to the number of isolates in that region's smaller cohort (typically the nonhuman group). These size-matched, bootstrapped datasets were then used to visualize the joint density of the virulence score (x-axis) and the AMR metric (y-axis) as contour plots, generated in Python (v3.10.12). Final figure panels for publication were assembled and annotated in Adobe Illustrator (v28.7.1).

**References**

X. Hua, Q. Liang, M. Deng, J. He, M. Wang, W. Hong, J. Wu, B. Lu, S. Leptihn, Y. Yu and H. Chen, BacAnt: A Combination Annotation Server for Bacterial DNA Sequences to Identify Antibiotic Resistance Genes, Integrons, and Transposable Elements, *Frontiers in Microbiology*, **12-2021** (2021).

D.M. Muloi, B.A. Wee, D.M.H. McClean, M.J. Ward, L. Pankhurst, H. Phan, A.C. Ivens, V. Kivali, A. Kiyong’a, C. Ndinda, N. Gitahi, T. Ouko, J.M. Hassell, T. Imboma, J. Akoko, M.K. Murungi, S.M. Njoroge, P. Muinde, Y. Nakamura, L. Alumasa, E. Furmaga, T. Kaitho, E.M. Öhgren, F. Amanya, A. Ogendo, D.J. Wilson, J.M. Bettridge, J. Kiiru, C. Kyobutungi, C. Tacoli, E.K. Kang’ethe, J.D. Davila, S. Kariuki, T.P. Robinson, J. Rushton, M.E.J. Woolhouse and E.M. Fèvre, Population genomics of Escherichia coli in livestock-keeping households across a rapidly developing urban landscape, *Nature Microbiology* **7** (2022), pp. 581-589.

B.D. Ondov, T.J. Treangen, P. Melsted, A.B. Mallonee, N.H. Bergman, S. Koren and A.M. Phillippy, Mash: fast genome and metagenome distance estimation using MinHash, *Genome Biology* **17** (2016), p. 132.

H.A. Thorpe, R. Booton, T. Kallonen, M.J. Gibbon, N. Couto, V. Passet, S. López-Fernández, C. Rodrigues, L. Matthews, S. Mitchell, R. Reeve, S. David, C. Merla, M. Corbella, C. Ferrari, F. Comandatore, P. Marone, S. Brisse, D. Sassera, J. Corander and E.J. Feil, A large-scale genomic snapshot of Klebsiella spp. isolates in Northern Italy reveals limited transmission between clinical and non-clinical settings, *Nature Microbiology* **7** (2022), pp. 2054-2067.

K.L. Wyres, M.M.C. Lam and K.E. Holt, Population genomics of Klebsiella pneumoniae, *Nature Reviews Microbiology* **18** (2020), pp. 344-359.
