# Supplementary material for: Eco‐Geography Reverses Dominant AMR Reservoirs in Klebsiella pneumoniae: Integron‐Rich Mobilomes and Cross‐Niche Connectivity
Source: Adv Sci (Weinh). 2026 May 6;13(42):e75537. doi: 10.1002/advs.75537 (PMC13336099; doi:10.1002/advs.75537)
Supplement: Supplementary file 2 — Supporting File 2: advs75537‐sup‐0002‐SuppMat2.docx. [file ADVS-13-e75537-s006.docx]

**Supplementary Materials**

**Eco-geography reverses dominant AMR reservoirs in *Klebsiella pneumoniae:* integron-rich mobilomes and cross-niche connectivity**

Hui Lin,^1^† Biao Tang,^2^† Hao Xu,^3^† Jiajie Qian,^4^† Feifan Shi,^1,5^ Junhui Zhao,^6^ Xiaorui Mao,^2^ Xiaohe Hu,^2^ Ruishan Liu,^3^ Wenhong Liu,^7^ Xiawei Jiang,^7^* Beiwen Zheng,^3,8,9^* Guoping Zhao ^2,10^

^1^ State Key Laboratory for Quality and Safety of Agro-Product, Zhejiang Provincial Key Laboratory of Agricultural Microbiomics, Institute of Environment, Resource, Soil and Fertilizer, Zhejiang Academy of Agricultural Sciences, Hangzhou, PR China

^2^ Key Laboratory of Systems Health Science of Zhejiang Province, School of Life Science, Hangzhou Institute for Advanced Study, University of Chinese Academy of Sciences, Hangzhou, PR China

^3^ State Key Laboratory for Diagnosis and Treatment of Infectious Diseases, National Clinical Research Center for Infectious Diseases, National Medical Center for Infectious Diseases, Collaborative Innovation Center for Diagnosis and Treatment of Infectious Diseases, The First Affiliated Hospital, Zhejiang University School of Medicine, Hangzhou, PR China

^4^ Department of Gastrointestinal Surgery, The First Affiliated Hospital, College of Medicine, Zhejiang University, Hangzhou, PR China

^5^ Xianghu Laboratory, Hangzhou, PR China

^6^ Department of Clinical Laboratory, The Second Affiliated Hospital of Wannan Medical College, Wuhu, PR China

^7^ School of Basic Medical Sciences, Zhejiang Chinese Medical University, Hangzhou, PR China

^8^ Yuhang Institute of Medical Science Innovation and Transformation, Hangzhou, PR China

^9^ Jinan Microecological Biomedicine Shandong Laboratory, Jinan, PR China

^10^ Department of Microbiology, School of Life Sciences, Fudan University, Shanghai, PR China

† Contributed equally: Hui Lin, Biao Tang, Hao Xu, and Jiajie Qian.

***** Corresponding authors: Beiwen Zheng and Xiawei Jiang.

E-mail: zhengbw@zju.edu.cn; xiaweijiang@zcmu.edu.cn.

**This PDF file includes:**

Figures. S1 to S10

Table S1

Legends for Data S1 to S10

**Other Supplementary Materials for this manuscript include the following:**

Data S1 to S10

Supplementary Figures

Figure S1.


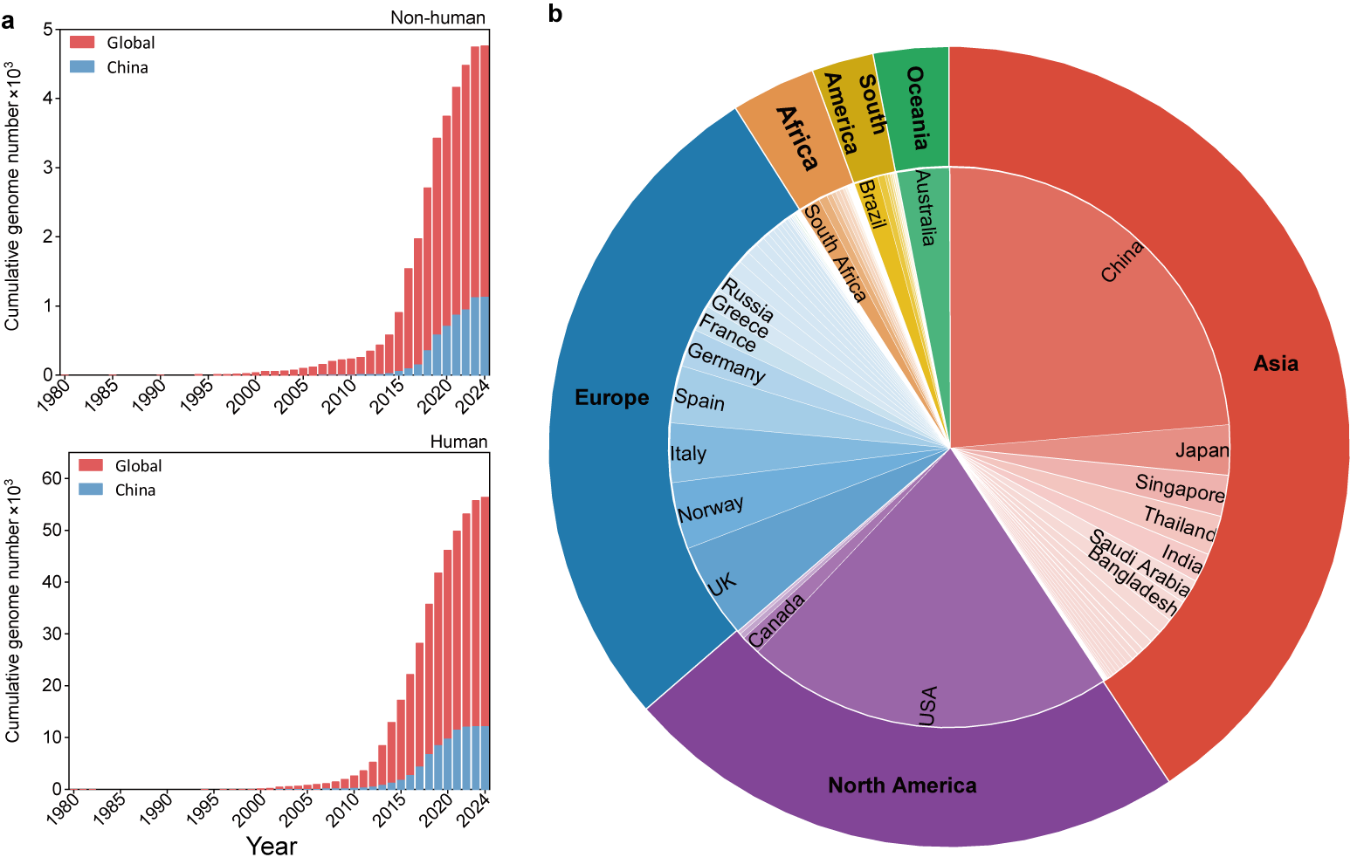


Figure S1. Temporal growth and global distribution of publicly available *Klebsiella pneumoniae* genomes. a, Changing trend of publicly available *K. pneumoniae* genome number over time, stratified by source (human and nonhuman) and compared between China and the global dataset. b, Distribution of genomes by country group or geographic region (pie chart)

Figure S2.


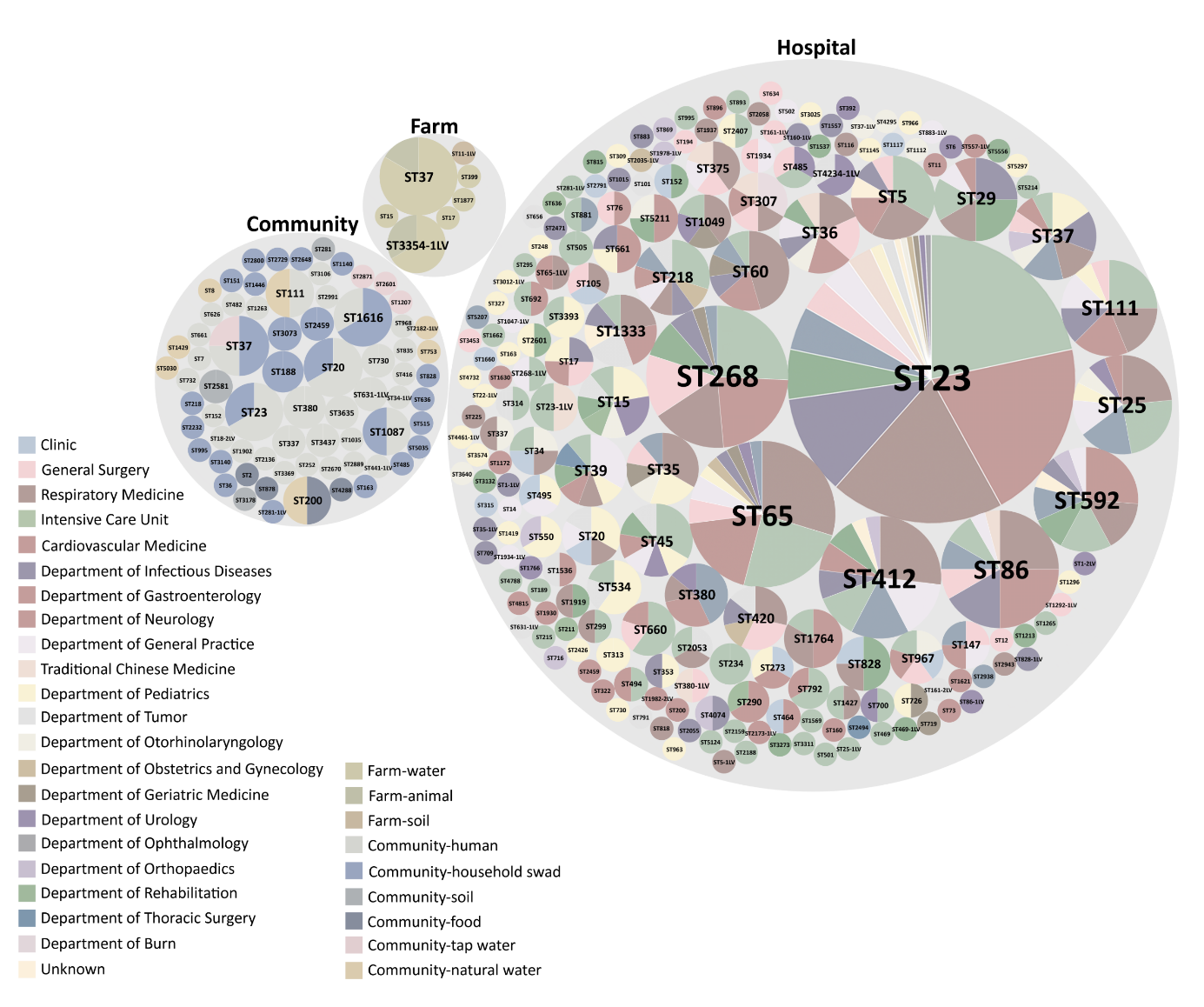


Figure S2. Species lineages of 820 *Klebsiella pneumoniae* isolates and their distribution across different sources. Sequence type (ST) composition colored by source types. Outer and inner circles represent setting types and STs, respectively. Larger circles indicate a greater number of isolates.

**Figure S3.**


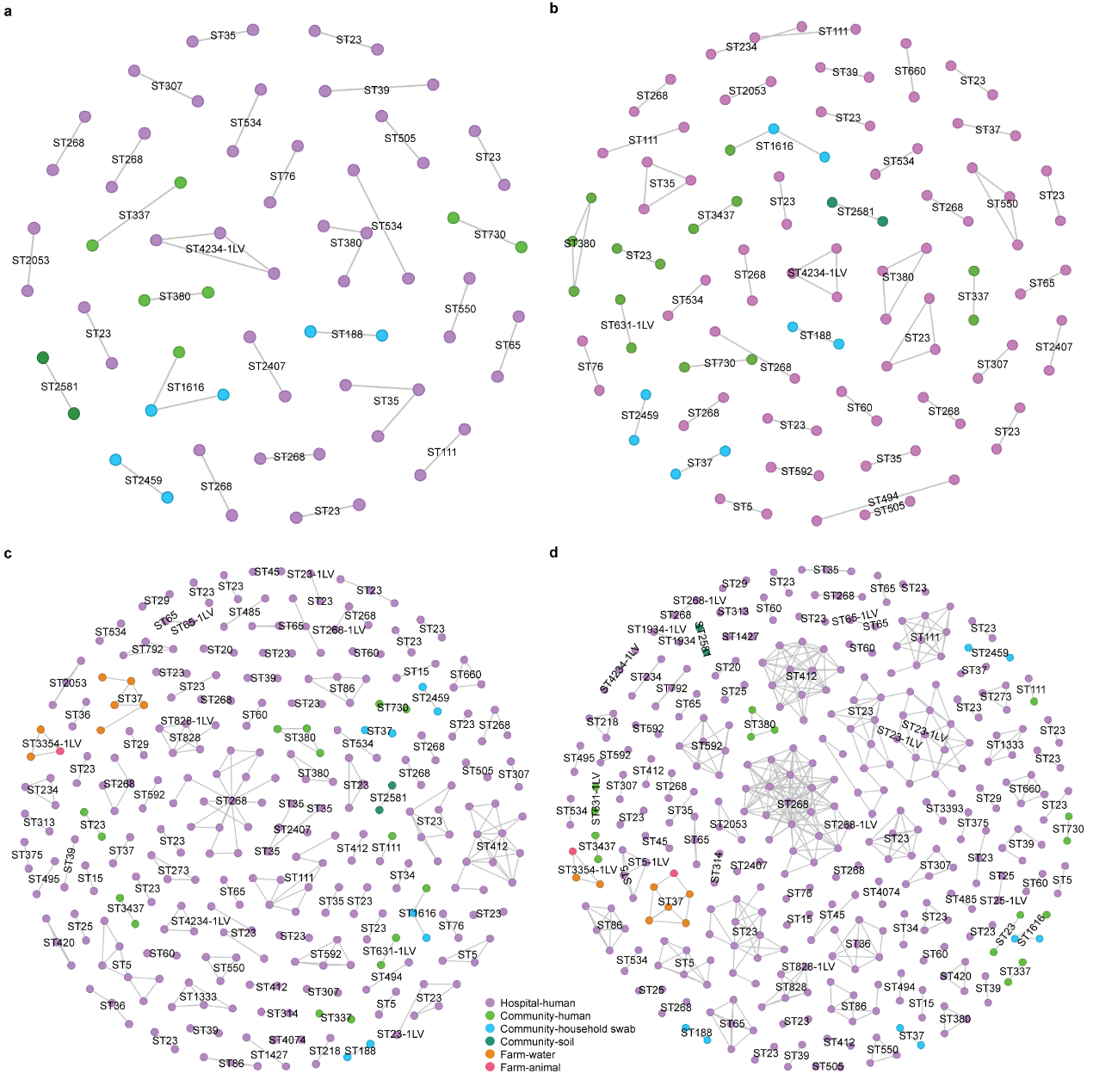


**Figure S3. Force-directed Fruchterman–Reingold layout visualizing clonal dissemination based on core genome single-nucleotide polymorphism (SNP) distances.** Each node represents a sequence type (ST), with colors indicating the source of the isolates. Edges between nodes represent genetic relatedness on the basis of SNP distances. Panels show dissemination patterns at varying SNP thresholds: **a,** 10 SNPs; **b,** 20 SNPs; **c**, 80 SNPs; **d**, 100 SNPs.

**Figure S4.**


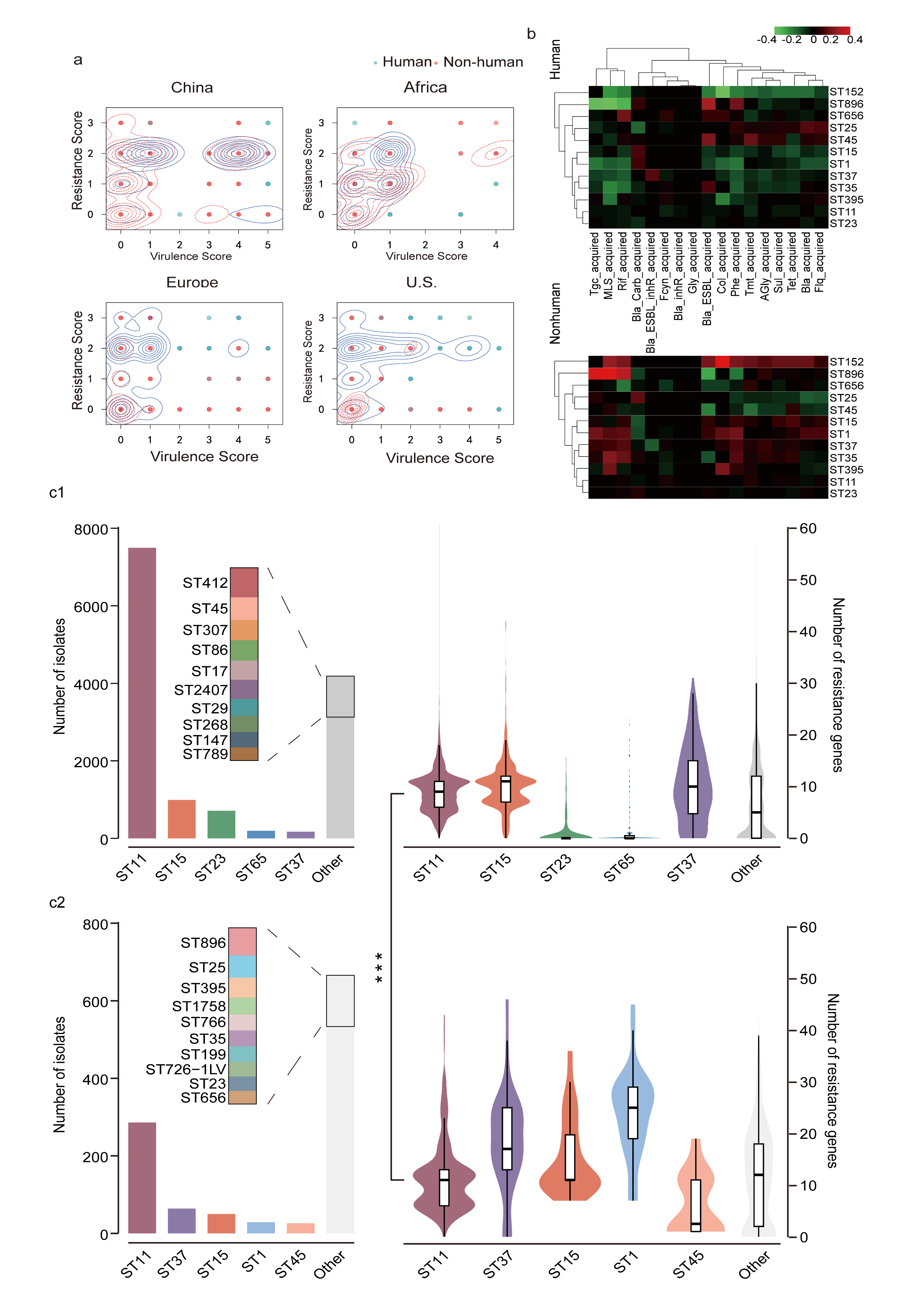


**Figure S4. Antimicrobial resistance (AMR)-virulence traits across human and nonhuman niches. a,** Density contour analysis showed the joint distribution of AMR and virulence scores across niches and regions. Blue and red contours denote human and nonhuman isolates, respectively. **b,** Heatmaps display differences in AMR gene profiles for specific sequence types (STs) between human and nonhuman sources based on global data. Bias scores for specific STs were calculated to indicate whether nonhuman or human isolates tend to enrich certain AMR classes. Positive bias scores represent enrichment, whereas negative scores indicate depletion, with color intensity reflecting the strength of the bias. Bias scores=$X-\frac{Human+non-human}{2}$, where X represents either the human or nonhuman index. **c,** ST composition and ARG profiles of isolates from China. **c1,** Human isolates showing the five most prevalent STs and the top 10 STs among the remaining STs (left), and the distribution of ARG numbers across these STs (right). **c2,** Nonhuman isolates showing the same analyses. Human- and nonhuman-derived ST11 isolates differed significantly in ARG counts (*P* < 0.001).

**Figure. S5.**


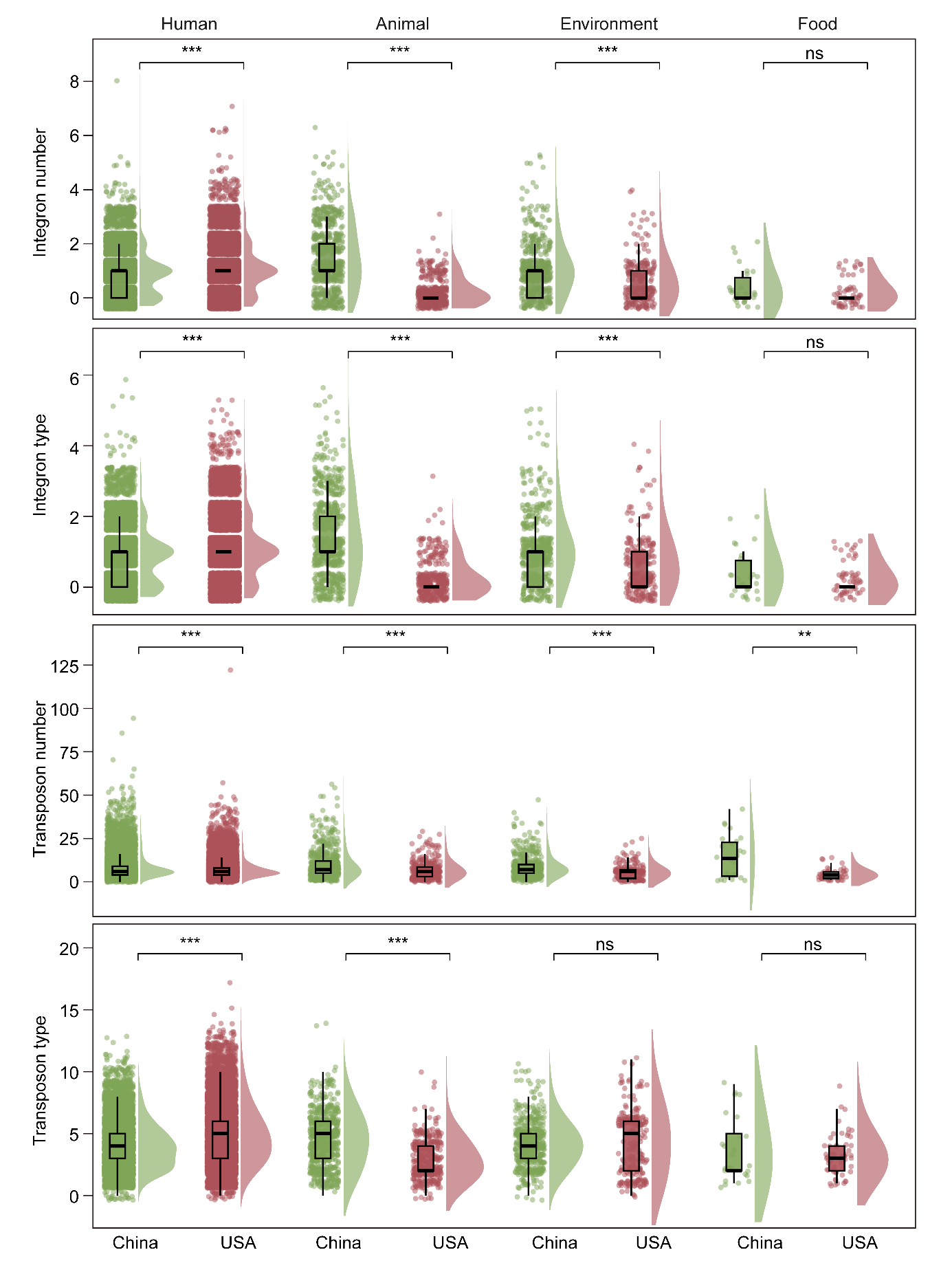


**Figure. S5.** **Country contrasts in mobile genetic elements (MGEs) metrics by source.** Violin plots compare China subset (green) versus the U.S. subset (red) for four MGEs metrics (integron number, integron type richness, transposon number, and transposon type richness) within each nonhuman (animal, environment, food)/human source category. Brackets denote Mann-Whitney U tests (*** *P* < 0.001; ** *P* < 0.01; *ns*, not significant). China-U.S. differences are significant for human, animal, and environment in integron number and integron type, but not for food; transposon number differs for human, animal, and environment, while transposon type differs for human and animal only, with no difference for environment or food.

**Figure S6.**

**
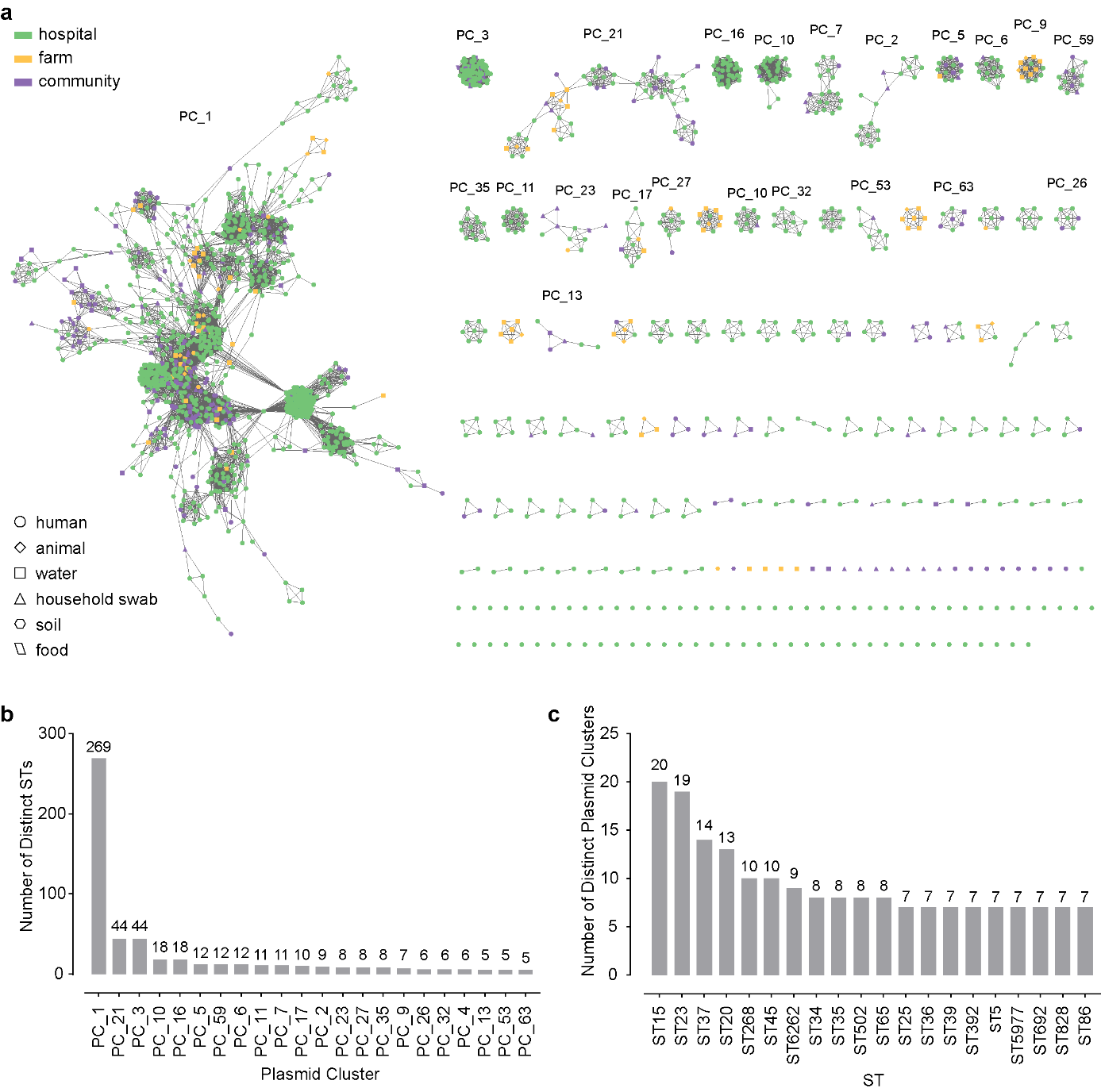
**

**Figure. S6. Plasmid sharing networks and their distribution across sequence types and ecological settings. a,** Plasmid similarity network based on single-linkage clustering. Nodes represent individual plasmids, and edges connect pairs with Mash distance ≤ 0.05. Node colors indicate the isolation setting (green: hospital; yellow: farm; purple: community), while node shapes denote the host category (circle: human; diamond: animal; square: water; triangle: household swab; hexagon: soil; parallelogram: food). The large interconnected component represents plasmid cluster 1 (PC_1), a highly promiscuous plasmid cluster spanning diverse hosts and environments, while smaller clusters and singletons are arranged to the right. **b,** Host range distribution of plasmid clusters (PCs). The histogram displays the top 22 PCs that span ≥5 distinct sequence types (STs), ranked by the number of STs they encompass. **c,** Plasmid diversity within sequence types (STs). The histogram shows the top 20 STs carrying ≥7 distinct plasmid clusters, ranked by PC count.

**Figure. S7.**


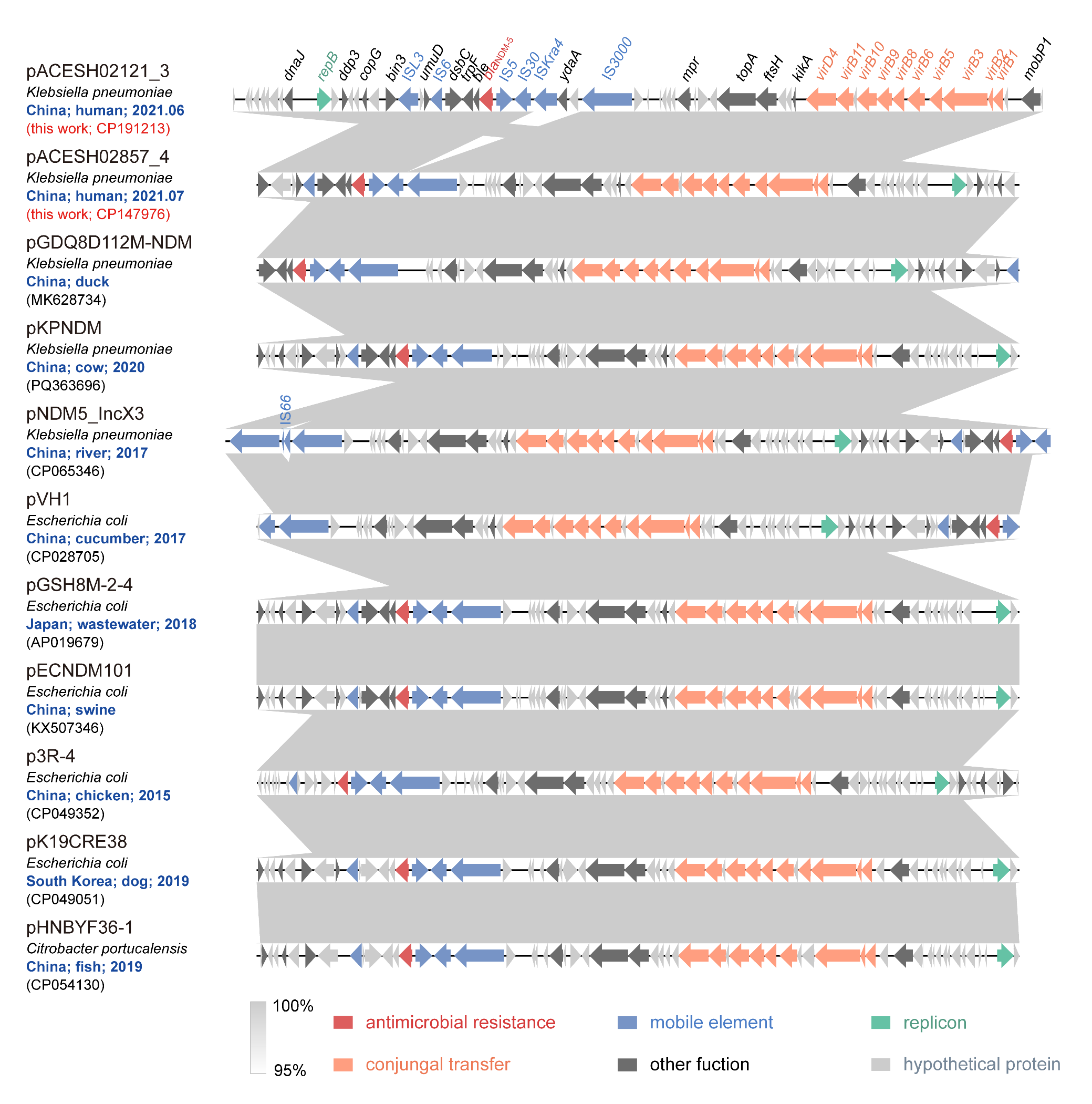


**Figure. S7. Genetic environment analysis of *bla*_NDM-5_-positive plasmids.** Comparison of IncX3 plasmids from this study (pACESH02857 and pACESH02121) and representative human, chicken, and sewage plasmids from GenBank.

**Figure. S8.**


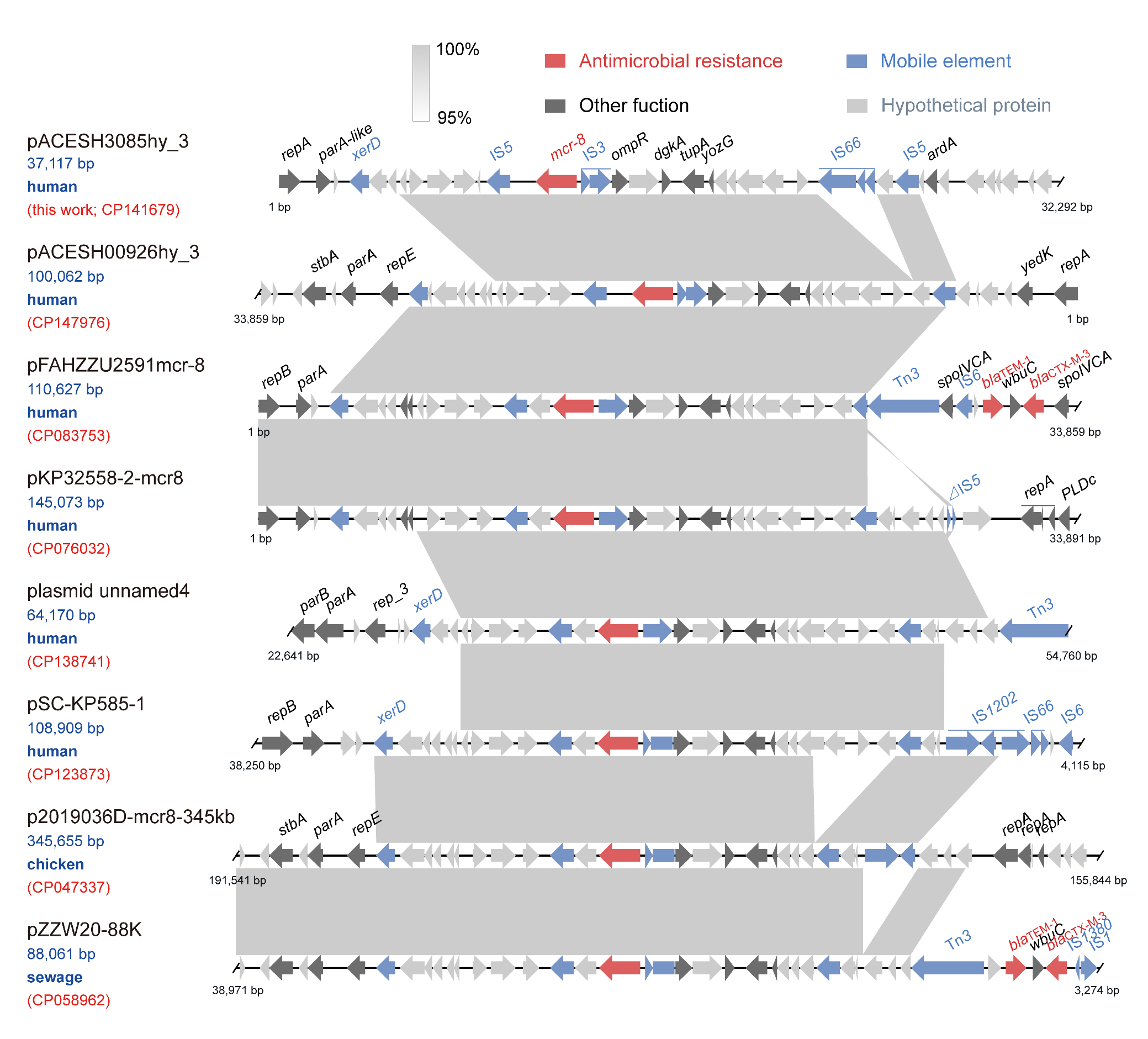


**Figure. S8. Comparison of *mcr-8.2* carrying plasmids**. There are pACESH00926hy_3 [110.6 kb] and pACESH03085hy_3 [37.1 kb] identified in this study and other *mcr-8.2* plasmids available in GenBank

**Figure. S9**


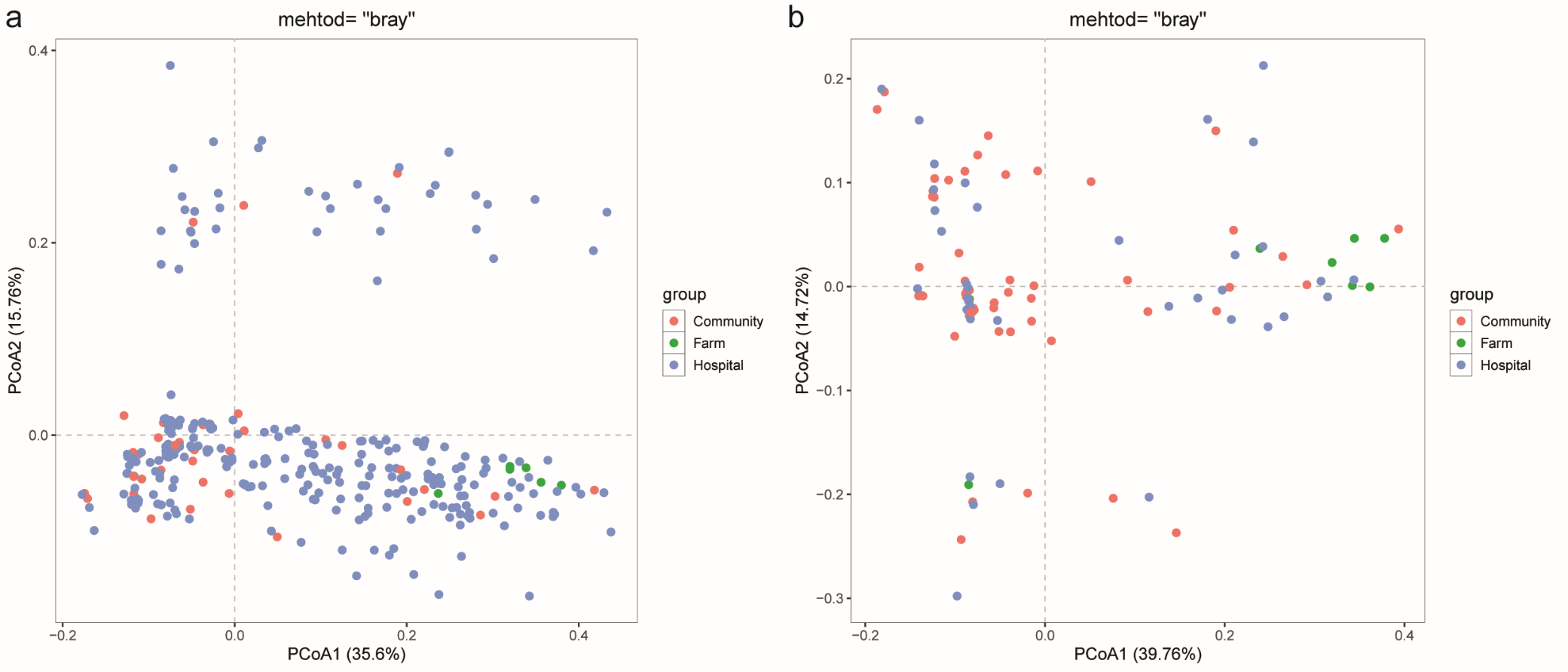


**Figure. S9 Principal coordinates analysis (PCoA; Bray-Curtis) of antimicrobial resistance (AMR) gene composition (mutation-mediated resistance excluded) in *Klebsiella pneumoniae* population.** Points are coloured by setting (hospital, community, farm). **a**, Full dataset (*n* = 820). **b**, June 2021 subset (*n* = 170), confirming the same separation under a single-month window. Bray-Curtis dissimilarity was used for the analysis. No clear setting-specific separation of AMR gene profiles was observed.

**Figure. S10.**


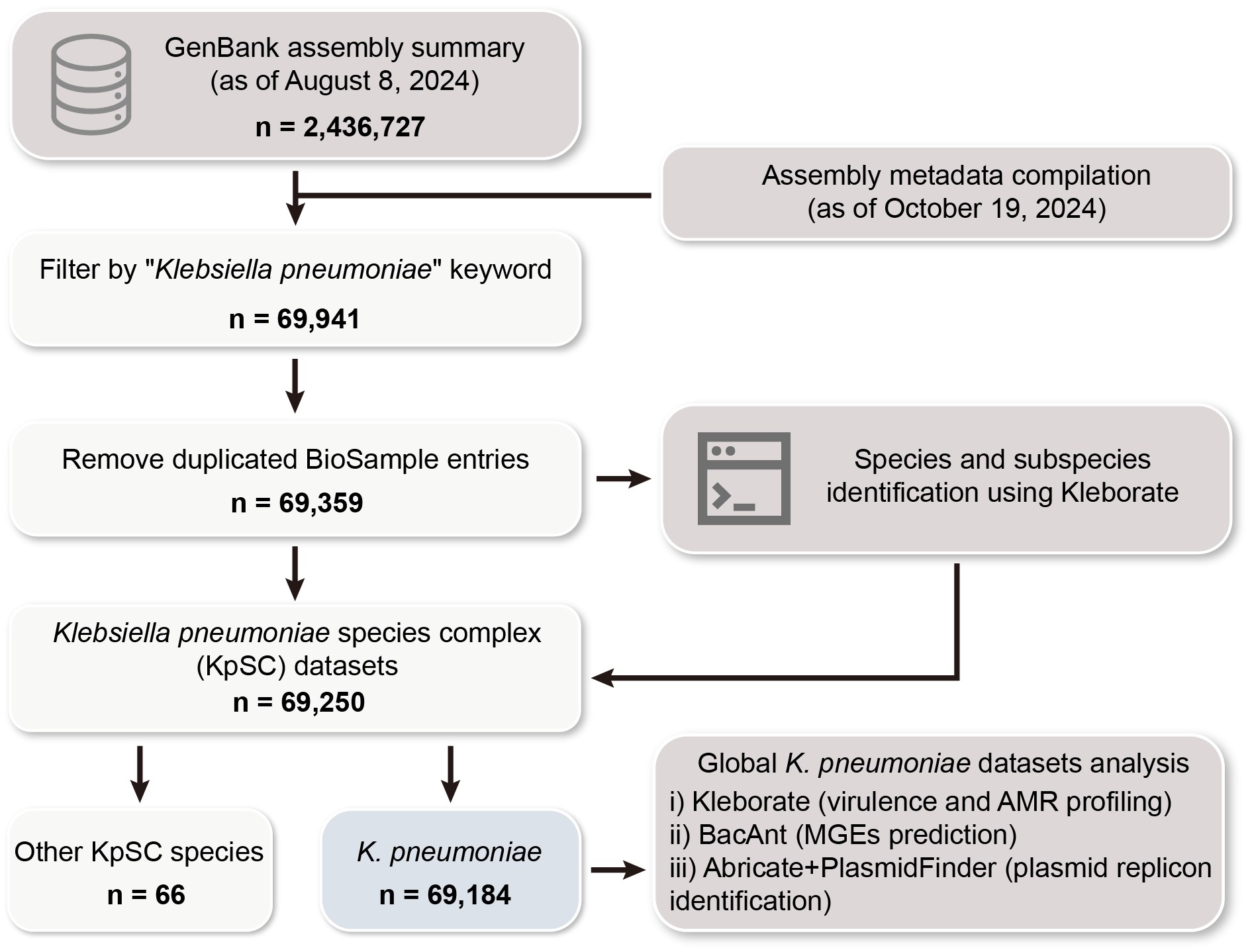


**Figure. S10. Global genome dataset construction and quality control workflow for the *Klebsiella* pneumoniae species complex (KpSC).** A comprehensive genome dataset was retrieved from the NCBI GenBank Assembly database as of August 8, 2024 (*n* = 2,436,727). Metadata (including isolation date, host, and geographic location) were independently compiled from NCBI on October 19, 2024, without expanding the genome collection. Genomes annotated with the keyword K. pneumoniae were selected (*n* = 69,941), followed by removal of duplicated BioSample entries to retain a single representative genome per BioSample (*n* = 69,359). Species and subspecies identification was performed using Kleborate, excluding 109 non-KpSC genomes and yielding a final dataset of 69,250 KpSC genomes. This dataset comprised 69,184 K. pneumoniae genomes and 66 genomes from other KpSC species. The finalized K. pneumoniae dataset was subsequently used for global genomic analyses.

Supplementary Table

Table S1. Comparison of genotypic and phenotypic antimicrobial resistance (AMR) profiles and mobile genetic elements (MGEs) among Pingguo *K. pneumoniae* isolates from different hosts.

| **Host** |  | **Isolate** | **AMR** | | | **MGEs** | | | |
| --- | --- | --- | --- | --- | --- | --- | --- | --- | --- |
|  |  |  | **Genotypic** | | **Phenotypic** | **Transposon** | | **Integron** | |
|  |  | Counts | Class number | Gene number | Class number | Class  number | Gene number | Class  number | Gene number |
| **Human** | Total | 755 | 1.94 | 2.81 | 1.92 | 2.66 | 7.89 | 0.25 | 0.25 |
|  | Hospital patients | 712 | 2.02 | 2.93 | 2.00 | 2.66 | 7.71 | 0.26 | 0.26 |
|  | Community human | 43 | 0.70 | 0.77 | 0.67 | 2.53 | 10.84 | 0.07 | 0.07 |
| **Nonhuman** | Total | 65 | 3.05 | 5.71 | 4.06 | 3.14 | 7.82 | 0.49 | 0.49 |
|  | Community nonhuman* | 51 | 1.98 | 2.90 | 2.41 | 2.75 | 7.08 | 0.18 | 0.18 |
|  | Farm animal | 2 | 7.50 | 17.00 | 9.50 | 5.00 | 10.00 | 1.50 | 1.50 |
|  | Farm environment | 18 | 6.83 | 15.75 | 10.17 | 4.50 | 10.58 | 1.67 | 1.67 |

***** Isolates collected from environments, food, or animals.

Supplementary Datasets

Data S1 Metadata summary of all samples collected in Pingguo.  Excel Sheet 1, hospital samples. Excel Sheet 2, community samples. Excel Sheet 3, farm samples. Metadata include Sample ID, culture medium (plate type), sampling date, sample type, source, and related information.

Data S2 Kleborate analysis results of 917 *Klebsiella pneumoniae* species complex and their metadata collected in Pingguo.

Data S3 Antimicrobial susceptibility test results of 917 *Klebsiella pneumoniae* species complex. Excel Sheet 1, Minimum inhibitory concentrations (MICs, μg·mL^-1^) are shown for each isolate (rows) against a panel of antibiotics (columns). Excel Sheet 2, the corresponding categorical susceptibility results (0=susceptible; 1=resistant) based on the interpretive criteria described in the Methods. Excel Sheet 3, antibiotic abbreviations with full names.

Data S4 Raw data of SNP distances for 820 *Klebsiella pneumoniae* isolates. Excel Sheet 1, complete pairwise SNP distance matrix. Excel Sheet 2, isolate pairs extracted from Excel Sheet 1 with SNP ≤ 100, annotated with sample source and sequence type. Excel Sheet 3: isolate pairs extracted from Excel Sheet 1 with SNP ≤ 10.

Data S5 Kleborate and MGEs analysis results for 820 *Klebsiella pneumoniae* isolates collected in Pingguo.

Data S6 Accession list for 69,184 global *Klebsiella pneumoniae* genomes and selected summary results from Kleborate and mobile genetic element analyses.

Data S7 A collections of non-redundant, high quality *K. pneumoniae* genomes for China, the U.S., Europe, and Africa.

Data S8 Comparison of genotypic antimicrobial resistance (AMR) profiles of global *Klebsiella pneumoniae* using different datasets. Excel Sheet 1, regional and source-specific AMR profiles in different genome datasets. Excel Sheet 2, Human vs. non-human comparisons of virulence and AMR metrics across regions (mean values, p-values, Cliff_delta data, and effect sizes).

Data S9 Correlation analysis of antimicrobial resistance (AMR) gene and class burdens with antibiotic consumption. Excel Sheet 1, antibiotic consumption (DDDs) by country, year, and antibiotic class (data source: DOI 10.1073/pnas.2411919121). Excel Sheet 2, dataset used for correlation analysis between AMR burdens and annual antibiotic consumption. Excel Sheet 3, correlation analysis results.

Data S10 Permutation test results for gauging whether different sequence types (STs) were non-randomly distributed between sources.
